# A Dimensionless Empirical Model to Predict Heat Transfer Coefficients for Cooling High‐Moisture Meat Analog with Rectangular Dies

**DOI:** 10.1111/1750-3841.70366

**Published:** 2025-07-05

**Authors:** Caleb E. Wagner, Leon Levine, Girish M. Ganjyal

**Affiliations:** ^1^ School of Food Science Washington State University Pullman Washington USA; ^2^ Leon Levine & Associates Albuquerque New Mexico USA

**Keywords:** extrusion, heat transfer, meat analog, protein, texturization

## Abstract

**ABSTRACT:**

An empirical dimensionless relationship useful for estimating heat transfer coefficients during continuous cooling of high moisture meat analog (HMMA) in rectangular linear cooling dies is described here. This information is essential for designing better cooling dies, which is timely since the cooling rate has recently been demonstrated to be important in controlling the quality of HMMA. Wheat‐based HMMA was extruded as per an experimental design that varied cooling media temperature (36–72°C), product mass flow rate (2.7–4.5 kg/h), and die aspect ratios (a* = 0.28 or 0.45). In situ product and cooling media temperatures and mass flow rates were measured continuously using penetrative thermocouples and flow meters, respectively. Dimensional scaling of the underlying differential equations governing heat transfer for this system demonstrated a relationship between dimensionless Nusselt ($\bar{\mathrm{Nu}}$), Graetz (Gz^−1^), and conduit aspect ratio (a*) numbers. Values for these numbers derived from the described experimental data were fit via nonlinear regression to a dimensionless model of the form $\bar{\mathrm{Nu}}$ = A∙(Gz^−1^)^B^∙(C+D∙[1−a*]^E^) with a high degree of quality (root mean square error = $\bar{\mathrm{Nu}}$ ± 4.9, *p* < 0.0001). The resulting fit parameters were deemed reasonable given that the model was logically bound within theoretical Nusselt number limits. The model documented here allows for the estimation of heat transfer coefficients relevant to HMMA cooling dies, while also illustrating the effect of altering cooling die aspect ratios and heat exchanger lengths as would be considered when scaling up an HMMA process.

**Practical Application:**

Carefully balanced cooling rates are essential for producing whole‐cut type meat analogs with a desirable texture and fibrous quality at an economically feasible scale. This work presents a useful model for estimating the required cooling rates of whole‐cut type meat analogs as a function of key processing criteria such as production rate and product size. Models like this would be useful for engineers and scientists attempting to optimize their meat analog processes, with such efforts ultimately resulting in reduced costs and improved quality associated with meat analog production.

## 1 Introduction

A major issue associated with the commercialization of high‐moisture meat analogs (HMMA) is scaling up the process such that good quality products can be manufactured at price parity (or better) relative to animal meat (Plattner 2020). More specifically, designing the cooling die geometry to accommodate commercial‐scale volumes while maintaining a target cooling rate is the most significant processing bottleneck associated with HMMA scale‐up. When producing HMMA, hydrated (>40% wet‐basis) protein‐rich doughs are heated to enable unfolding, a flowable liquid state, and subsequent chemical rearrangement of the polymeric proteins (Chen et al. 2011; Richter et al. 2023; Schmid et al. 2022; Ubbink and Muhialdin 2022). The thermally transformed protein melt then flows into a cooling die, where it is transformed into a meat analog with a whole muscle‐like appearance (Ryu 2020; Twombly 2020). Recent literature has demonstrated that the shear and heat transfer rates applied in the cooling die dictate HMMA structure and quality (Guyony et al. 2022; Kaunisto et al. 2024; Köllmann et al. 2023; Sandoval Murillo et al. 2019; Wagner et al. 2024). Despite the demonstrated importance of the HMMA cooling die, there is a lack of fundamental engineering data relevant to operating, designing, or scaling commercially sized dies. Such data should include, at a minimum, estimates of the heat transfer coefficients that are essential for predicting cooling rates for a given design.

Convective heat transfer coefficients (conventionally denoted h) are required to solve applied heat transfer problems, such as calculating heat transfer area requirements or numerically estimating temperature distributions. However, measuring h for most real systems is a nontrivial task since material thermophysical properties, duct geometry, product velocity, applied temperature differential, and how the product‐to‐heat transfer media interface all affect the value of h (Incropera et al. 2007; Toledo 1999). Compounding this complexity is the fact that it can be difficult to collect such data for many closed processes. Instead of attempting to measure values of *h* directly, it is common practice to utilize empirical dimensionless relationships (often referred to in the engineering field as dimensionless correlations) to first calculate a dimensionless heat transfer coefficient (i.e., Nusselt number), which is subsequently converted into an estimate of h after reintroducing dimensional quantities. General relationships between dimensionless variables are often determined logically via dimensional analysis (e.g., Buckingham Pi‐theory) or analytically by rendering dimensionless the differential equations governing specific transport phenomena (e.g., dimensional scaling). Once the pertinent dimensionless variables and their general relationships to each other are established in this theoretical context, a model can be fit to empirical data whose input and output variables have been expressed as the predetermined dimensionless numbers. There are numerous such relationships in the literature since they are usually specific to a limited range of boundary conditions. One such relationship possibly applicable to HMMA is Nu = f(Re, Pr, D/L), which is useful for characterizing laminar flow heat transfer in very wide slit dies. In this relationship, Nu, Re, and Pr are (respectively) the dimensionless Nusselt, Reynolds, and Prandtl numbers, while the ratio D/L is a characteristic conduit dimension (D) over the same conduit's axial length (*L*) (Incropera et al. 2007; Toledo 1999). The product of Re, Pr, and D/L is often expressed as a Graetz number (Gz), which is intuitively described for closed conduit laminar fluid flow as the ratio of heat advected parallel to the product flow to the heat conducted perpendicular to the flow (Haase et al. 2015). To our knowledge, no attempts to measure heat transfer coefficients applicable to an HMMA cooling process have been undertaken outside of a limited set of dimensional overall heat transfer coefficients published by Lee et al. (2005).

A simple dimensionless model useful for estimating h for HMMA made in linear rectangular cooling dies is documented in this work, which would be useful to engineers and scientists with an introductory understanding of transport phenomena. This data is relevant to the practical design or analysis of new or existing cooling dies, respectively. Such data could also be used to create more realistic simulations of heat transfer in HMMA dies. To our knowledge, this is the first time this fundamental information has been reported in the literature in an HMMA context.

## 2 Materials and Methods

### 2.1 Experimental Design, Materials, and Thermal Constant Definitions

**FIGURE 1:**
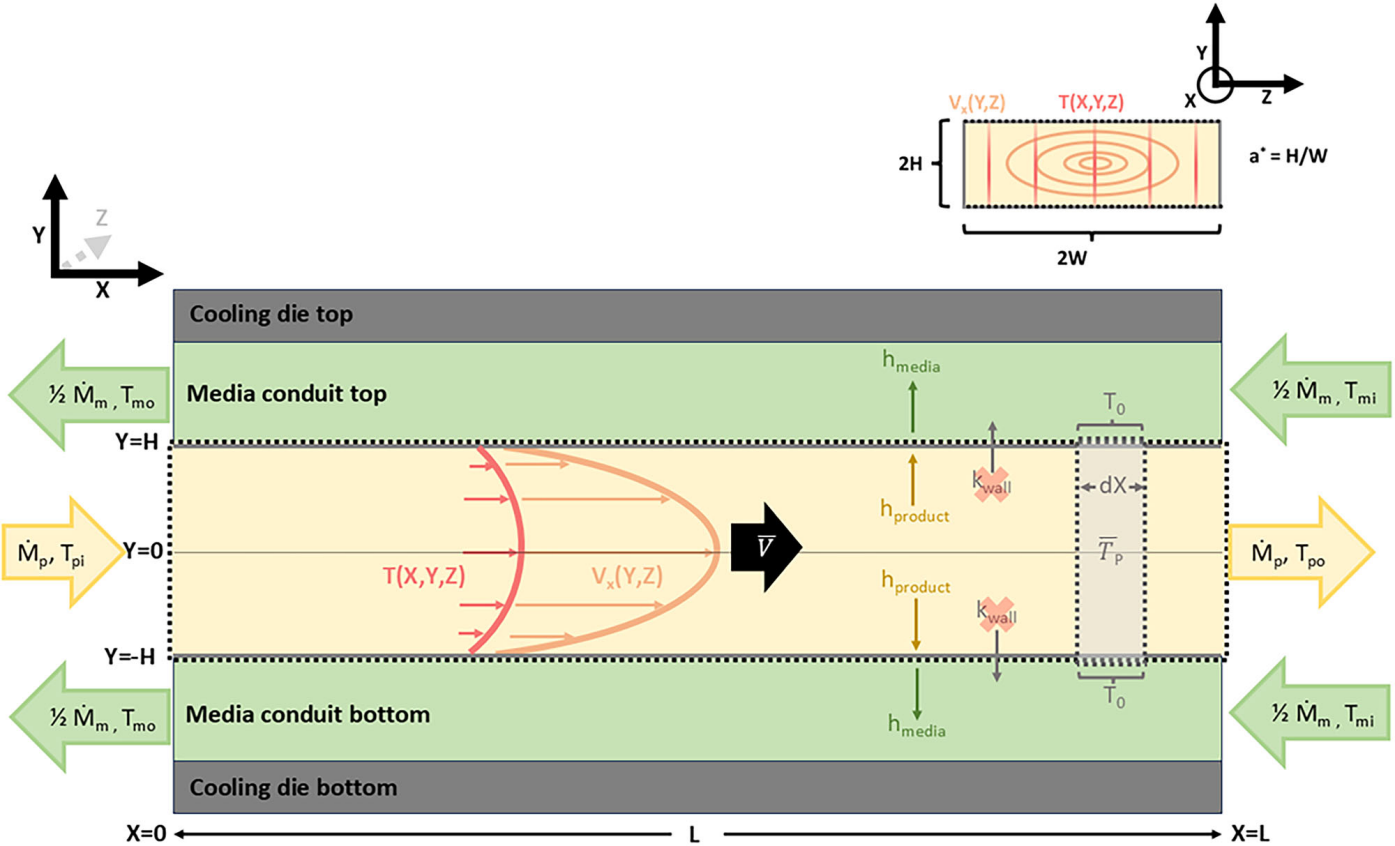
Relevant cooling die dimensions and variables needed for heat transfer calculations. The upper right‐hand corner shows an arbitrary cross‐sectional view of the product conduit with relevant dimensions and the definition of aspect ratio (a*). Note that a black dotted line indicates a boundary across which heat transfers out of the product system. The differential slice (dX) exemplifies an area where the bulk average product temperature (T̅_p_) and average periphery temperature (*T*_0_) would be derived from when calculating the local Nusselt number (Nu_x_) for any given X position.

**FIGURE 2:**
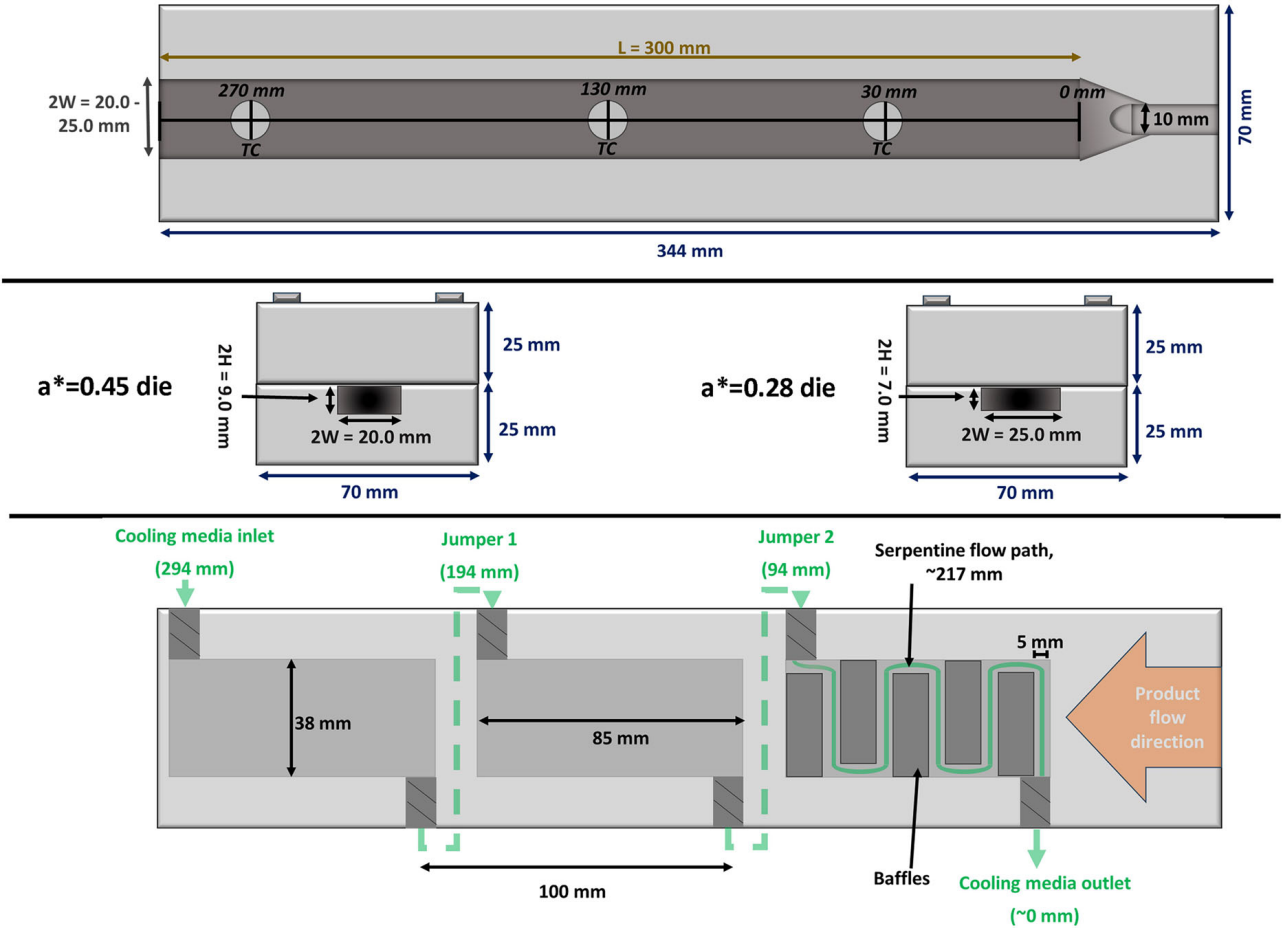
Cooling die configuration. The top and middle panels show the axial and transverse configuration of the product channel, respectively; the bottom panel shows a cutaway of the media side. All three discrete media conduits in each half of the die are identical, making for six discrete heat exchange areas over the entire length of the die. Each media conduit covers about 1/3 of the product channel length, and thus each section is referred to as a nominal 1/3, 2/3, or 3/3 progression of the die length.

**FIGURE 3:**
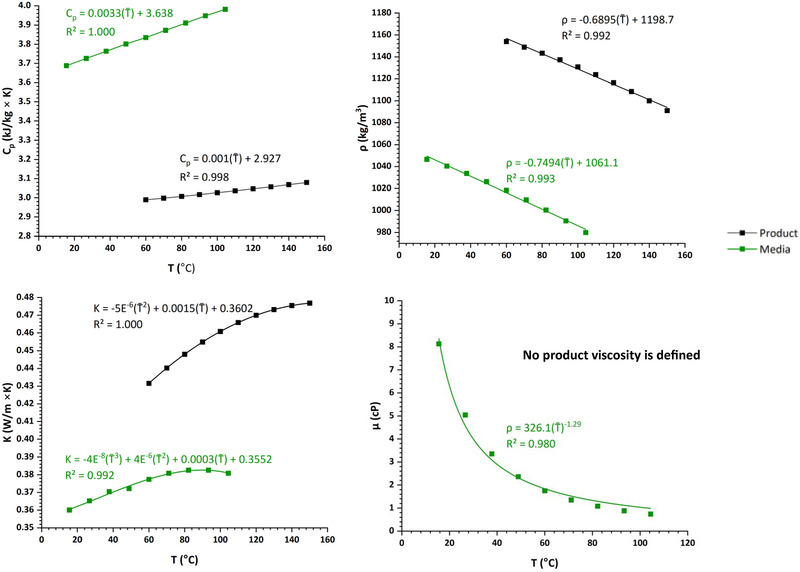
Material thermal properties used in all calculations. The temperature correction equation used in each of these calculations is shown near the corresponding curve. The variable quantity T̅ in each equation is taken as the bulk average temperature of a given fluid stream (i.e., |(*T*_i−_ *T*_o_)/2|).

The data collected in the study by Wagner et al. (2024) was supplemented with complementary experimentation to produce a combined data set covering a thorough and orthogonal (i.e., independent) range of product mass flow rates (2.7, 2.96, 3.6, 4.25, and 4.50 kg/h), cooling media temperatures (36.0, 41.3, 48.0, 54.0, 60.0, 66.7, and 72.0°C), and cooling die product conduit aspect ratios (a* = 0.28 or a* = 0.45). Figure 1 provides a visual overview of what variables were measured during experimentation or subsequently calculated. The exact same equipment (TSE 20/40 Twin Screw Extruder, Brabender GmbH & Co. KG, Duisburg, Germany; Type 15–37‐000 volumetric feeding system, Brabender Technologie, Mississauga, ON, Canada; model A40AJ‐A‐4–0‐2 penetrative thermocouples, MPI Morheat inc., Etobicoke, Ontario, Canada; 50% propylene glycol heat transfer media; and insulated die assembly), wheat protein isolate (Arise® 8200, MGP Ingredients, Atchison, KS, USA), and data collection procedures (temperature and mass flow rate measurements, calibration procedures, etc.) described in Wagner et al. (2024) were used to collect this complementary data. The cooling die design details are provided in Table 1 and Figure 2. The density (*ρ*), heat capacity (*C*_p_), thermal conductivity (*k*), and viscosity (*µ*) constants used in calculations here are expressed at the measured bulk average fluid temperature (T̅ = [*T*_i_+*T*_o_]/2), where *T*_i_ is a stream inlet temperature while *T*_o_ is a stream outlet temperature) for a given stream unless stated otherwise. Figure 3 documents the temperature correction functions used to calculate these constants.

**TABLE 1:** Relevant cooling die product and media side conduit dimensions used in calculations.

Cooling die side	Quantity	a* = 0.45	a* = 0.28
Product (entire die)	Channel dimensions (2W × 2H × L_p_, mm)	20.0 × 9.0 × 300.0	25.0 × 7.0 × 300.0
	Aspect ratio, *a* ^*^(*a* ^*^ = 2*H*/2*W*)	0.45	0.28
	Cross‐sectional area, *A*_c,p_ (mm^2^)	180	175
	Wetted perimeter, *P*_p_ (mm)	58	64
	Equivalent diameter, *D*_h,p_ (mm)^a^	12	11
	Heat transfer area, *A*_p_ (mm^2^)	12000	15000
Cooling media (per unit)^b^	Serpentine baffled channel dimensions (*W*_m,u_ × *H*_m,u_ × *L*_m,u_, mm)	5 × 12 × 217	5 × 12 × 217
	Cross‐sectional area, *A*_c,m_ (mm^2^)	60	60
	Wetted perimeter, *P*_m_ (mm)	34	34
	Equivalent diameter, *D*_h,m_ (mm)^a^	7	7
	Unbaffled channel dimensions (*W*_m,ub_ × *H*_m,ub_ × *L*_m,ub_, mm)^c^	38 × 12 × 85	38 × 12 × 85
	Effective heat transfer area, *A*_m_ (mm^2^)^c^	3230	3230

^a^ Calculated as $D_{h}\equiv \frac{4\cdot A}{P}$, where A is the cross‐sectional area and P is the wetted perimeter of a given conduit.

^b^ The media conduit is composed of 3 discrete and identical units plumbed in series for each of the top and bottom sections of the die. Thus, there are 6 total discrete media units in the die, and the provided heat transfer area must be scaled according to how much of the die is being examined in calculations (e.g., 1/3 of the die length contains 2 × 3230 mm^2^ of media side heat transfer area, 2/3 of the die contains 4 × 3230 mm^2^, etc.). Please see Figure 2 for a schematic of the cooling media conduit.

^c^ This would be the cooling media conduit unit dimensions and area available for heat transfer if the baffling were not in place (i.e., *W*_m,ub_ × *L*_m,u_).

### 2.2 Dimensionless Relationship Conception

The flow of HMMA melt cooling in a linear cooling die is well described as a Graetz‐Nusselt type problem: the steady state heat transfer of an enclosed, thermally developing yet hydrodynamically fully developed laminar flow advecting heat to a conduit wall. It is assumed that there is a constant wall temperature, no axial product heat conduction, and no viscous heat dissipation (Haase et al. 2015). However, since partial to full slip is occurring over a major portion of the product duct (Kaunisto et al. 2024; Wagner et al. 2024), it cannot be certain that the flow is hydrodynamically fully developed; even so, this assumption can be justified intuitively by noting that the product side Pr number for HMMA is very large (given a relatively high melt viscosity), such that the rate of velocity profile development greatly outpaces the thermal profile development (Hirbodi et al. 2022). Using this simplified framework, the heat transfer behavior of HMMA being cooled in linear dies can be modeled using dimensionless relationships inferred from dimensional scaling of the underlying differential equations governing energy transport.

Using the system definition provided in Figure 1 and the boundary conditions and assumptions provided in Tables 2 and 3, respectively, it can be shown (see Supporting Information data) that a useful dimensionless relationship to understand heat transfer of cooling HMMA product in a rectangular duct can be defined by Equation (1):

(1) $$\bar{\mathrm{Nu}}=f\left(\mathrm{Gz},a*\right),$$

where the aspect ratio (a*) is defined as the ratio of duct height over width (Figure 1), and the Graetz number (Gz) describes the ratio of heat transported via flow into a duct over the heat conducted away at the periphery of said duct (Haase et al. 2015). Calculating Gz generally requires a flowing fluid's density (*ρ*), heat capacity (*C*_p_), average velocity (*V̄*), coefficient of thermal conductivity (*k*), and environmental geometry (i.e., duct hydraulic diameter [*D*_h_] and duct length [*L*]) as inputs (Equation 2):

(2) $$\mathrm{Gz}\;\equiv \frac{\rho C_{p}\;\bar{V}\;{D_{h}}^{2}}{Lk}.$$

**TABLE 2:** Boundary conditions (BCs) of the problem described in Figure 1. Dimensional and dimensionless conditions utilized in the model development are shown.

BCs number	Dimensional BCs	Corresponding dimensionless BCs
1	$X=0,\;\;T{|}_{all\;Y}=T_{p,i}\;$ ^a^	⌣X=0,Θ=1 ^a^
2	$T_{0}<\bar{T}\leq T_{p,i}$	0<Θ≤1 ^b^
3	$X=L,\;\;T{|}_{all\;Y}=T_{p,o}\;$	⌣X=1,0<Θ<1 ^c^
4	$Y=\pm H,T=T_{0}\;$	⌣Y=±HDh,Θ=0

^a^ Implies that the entrance temperature is uniform across the die cross section (i.e., *T*|*_X_*_= 0_ = *T̅*|*_X_*_= 0_ = *T*_pi_).

^b^ Another result of BCs 1, with the added constraint that the product temperature in a given axial position can never be lower than the local wall temperature (i.e., *T*_b_ > *T*_0_).

^c^ A direct result of boundary conditions 1 and 2, with the added assumption that some cooling has occurred (i.e., *T*_po_ < *T*_pi_) and that *T*_po_ = T̅|_X = L_ > T_0_.

**TABLE 3:** Assumptions made to simplify the energy equation.

Assumption #	Assumption description	Energy term(s) affected
1	System is at steady state	$\frac{\partial T}{\partial t}=0$
2	Density is constant	$\frac{\partial P}{\partial T}=0$
3	Constant thermal conductivity	k = constant
4	Negligible viscous dissipation	$\mu \nabla^{2}\cdot V$
5	No vertical or horizontal velocity components	$V_{Y}=V_{Z}=0$
6	Negligible axial conduction	$\frac{\partial^{2}T}{\partial X^{2}}\approx 0$
7	No heat accumulation or generation term	q̇ = 0

Since heat transfer‐relevant dimensionless numbers such as Gz were often defined originally for tubes with circular cross sections, *D*_h_ was used in the dimensional scaling process here to adapt the numbers to the rectangular cross sections employed, as suggested by Warrier et al. (2002) and Suzzi and Lorenzini (2019). It is worth noting that the Gz value is often expressed as Gz^−1^ in heat transfer literature since the inverse term allows for a more intuitive interpretation of the value as a dimensionless length over which heat transfer is taking place; this convention will be maintained here.

The average Nusselt number ($\bar{\mathrm{Nu}}$) estimates the magnitude of convective to conductive heat transfer across the entire duct, and results from averaging the local Nusselt numbers (Nu_x_) over the length of the cooling die (see Supporting Information data) as suggested by various authors (Bird et al. 2007; Valentas et al. 1991). To be consistent with the Gz definition used here, Nu is defined with *D*_h_ as the characteristic dimension of the duct as well (Equation 3):

(3) $$\mathrm{Nu}\;\equiv \frac{hD_{h}}{k},$$

where *h* is the convective heat transfer coefficient of interest that can be extracted from a calculated Nu upon rearrangement of Equation (3).

### 2.3 Calculation of Heat Transfer Coefficients and Dimensionless Numbers

#### 2.3.1 Overall Heat Transfer Coefficients

An apparent overall heat transfer coefficient (*U*_p_) proportional to the quantity of heat transferred sensibly was calculated as per the derivation detailed by Lee et al. (2005) (Equation 4):

(4) $$U_{p}=\frac{{\dot{M}}_{p}C_{p,p}\Delta T_{p}}{A_{p}\;\Delta T_{\ln}},$$

where *Ṁ*_p_ is product mass flow rate, *C*_p,p_ is the product heat capacity, Δ*T*_p_ is the product temperature change (*T*_p,o_—*T*_p,i_), *A*_p_ is the product side heat transfer area, and Δ*T*_ln_ is the log mean temperature across the cooling die product and media streams (Equation 5):

(5) $$\Delta T_{\ln}=\frac{\left(T_{p,o}-T_{m,i}\right)-\left(T_{p,i}-T_{m,o}\right)}{\ln\left[\frac{\left(T_{p,o}-T_{m,i}\right)}{\left(T_{p,i}-T_{m,o}\right)}\right]},$$

where *T*_p,i_ and *T*_p,o_ are the product inlet and outlet temperatures, respectively, while *T*_m,i,_ and *T*_m,o_ are the analogous terms on the media side.

Note, the *U*_p_ term defined here is apparent as it does not account for any latent heat from phase changes or viscous dissipation that may be occurring in the product. The magnitude of the latent heat terms is unknown at present, and the viscous dissipation terms can be shown to be negligible even when assuming all of the pressure drop experienced by the extrudate is lost to friction (i.e., geometric expansion and contraction are not considered).

#### 2.3.2 Extraction of Product Side Convective Heat Transfer Coefficients

The summation of thermal resistances was used to extract the product side convective heat transfer coefficients from the calculated quantity *U*_p_ (Equation 4) as detailed in various reference texts (Geankoplis et al. 2018; Singh and Heldman 2014; Toledo 1999). In this scenario, the cooling die wall is analogous to a conductive slab, with convective heat transfer occurring on both sides of the slab. Accounting for the nonequal heat transfer areas of the product and media side conduits (Table 1) results in Equation (6):

(6) $$\frac{1}{U_{p}\;A_{p}}=\frac{1}{h_{p}A_{p}}+\frac{\Delta Y_{\mathrm{wall}}}{k_{\mathrm{wall}}A_{\mathrm{pm}}}+\frac{1}{h_{m\;}A_{m}},$$

where Δ*Y*_wall_ is the thickness of the die wall, *k*_wall_ is the thermal conductivity of the wall, *A*_pm_ is the average of the product and media side heat transfer areas, *A*_m_ is the media side heat transfer area, and *h*_p_ and *h*_m_ are the convective heat transfer coefficients of the product and media sides, respectively. The wall conductivity term can be neglected here since the wall conduction rate is much greater than the convection rate of either fluid stream, which is intuitively supported when considering that both streams are in laminar flow and the wall is composed of a thin body of highly conductive stainless steel. Applying this simplification and rearranging Equation (6), we arrive at an expression for *h*_p_ (Equation 7):

(7) $$h_{p}=\frac{1}{(1/U_{p)}-(A_{p}/(h_{m\;}A_{m}))}.$$

Application of Equation (7) requires an estimate of *h*_m_ on the cooling media side of the heat exchanger, which can be calculated from the media‐side Nusselt number (Nu_m_) using a rearranged version of Equation (3):

(8) $$h_{m}=\frac{N_{u,m\;}k_{m}}{D_{h,m}}\;.$$

The Nu_m_ input for Equation (8) was calculated by applying the dimensionless Seider–Tate model defined for laminar flow forced convection (Kakaç et al. 2012) to the analysis of the media side.

(9) $$\begin{array}{ccc} Nu_{m} & = & 1.86\;R{e_{m}}^{1/3}\;P{r_{m}}^{1/3}{\frac{D_{h,m}}{L_{m,u}}}^{1/3}\;{\left(\frac{\mu_{m}}{\mu_{\mathrm{wall}}}\right)}^{0.14}\\ & = & 1.86\;G{z_{m}}^{1/3}\;{\left(\frac{\mu_{m}}{\mu_{\mathrm{wall}}}\right)}^{0.14}, \end{array}$$

where *D*_h,m_ is the equivalent diameter and *L*_m,u_ is the length of one serpentine media conduit section (7 and 217 mm, respectively, Table 1). The media viscosity, *µ*_m_, is defined at the T̅ of the media, while *µ*_wall_ is the media viscosity defined at the average wall film temperature (*T*_wall_ = [T̅_p_ + T̅_m_]/2, where T̅_p_ and T̅_m_ are the bulk average temperatures of the product and media streams, respectively). Re_m_ is the cooling media Reynolds number, and Pr_m_ is the cooling media Prandtl number, the product of which (along with the geometric terms) can alternatively be defined as the cooling media Graetz number (Gz_m_). The quantity L_m,u_ is defined for just one discrete media section instead of an additive conduit length, since it reflects the actual conduit length available for thermal development within each media section. The *T*_wall_ temperature calculated that dictates the *µ*_wall_ term is somewhat speculative, but it should be realized that any error associated with such a calculation is minimized when it is raised to the 0.14 power. Equation (8) is valid only for Gz_m_^1/3^∙(*µ*_m_/*µ*_wall_)^0.14^ ≥ 2 as per Kakaç et al. (2012), a criterion that is satisfied here. Given that baffling was present in the media conduits, it was assessed if a cross‐flow correction (product side flow unmixed, cross‐flowing media side mixed) of the Δ*T*_ln_ values defined in Equation (5) would be necessary; this was not the case for the situation here, as the temperature effectiveness and Δ*T* ratio values were sufficiently small and large, respectively, to make the Δ*T*_ln_ correction factor ∼1 in all cases (Kakaç et al. 2012).

With a value of *h*_p_ found, a value for $\bar{\mathrm{Nu}}$ can be estimated by applying Equation (3). These values, along with values of Gz^−1^ averaged over equal lengths of the die, are used to build the dimensionless relationships inferred in Equation (1). The mechanics of actually modeling these variables are discussed in the next Section 2.4.

### 2.4 Building the Dimensionless Heat Transfer Model

Given the ubiquity of power‐law type relationships in many empirical models, one possible model structure for the dimensionless relationship inferred by Equation (1) would be as follows (Equation 10):

(10) $$\bar{\mathrm{Nu}}=A{\left(Gz^{-1}\right)}^{B}\left(C+D{\left[1-a*\right]}^{E}\right),$$

where A, B, C, D, and E would be parameters derived from nonlinear regression. The general parabolic structure of the a*‐containing term in Equation (10) (C+D∙[1−a*]^E^) was chosen since Nu ∝ a* relationships theoretically follow such a relationship for rectangular ducts (Shah and London 1978). The parabolic structure also has the benefit of allowing Equation (10) to be defined at a* = 0 or a* = 1, unlike potentially simpler model structures. To verify that Equation (10) can conceptually be such a simple superimposition of the Gz^−1^ and a* terms, experimental values of $\bar{\mathrm{Nu}}$ and Gz^−1^ were linearized using base 10 logarithms. Simple visual inspection of the resulting linear trend showed that there was a clear parallel partition across a* (see Supporting Information data), and thus that no interaction terms would be necessary. After justifying the model structure as such, experimental data were fit to Equation (10) using nonlinear fitting tools in OriginPro software (Version 2023b, OriginLab Corporation, Northampton, MA, USA). The only constraints imposed during fitting were that −1 ≤ *B* ≤ −1/3, while parameter *E* was incrementally increased in fixed values of 0.5 units (beginning at *E*  =  1) until the equation could be fit with a value of parameter *C* ≥ 1; this final value of *C* was ultimately factored out of the final fit equation to reduce the number of parameters, with the fitting algorithm being reran with fixed values of *C* = 1 and *E* = 2.5 such that representative precision and significance values for the remaining parameters could be provided.

The model built here applied Equation (10) to model the Nu ∝ Gz^−1^ relationship specific to the nominal first 1/3 (*L* = 94 mm), the nominal 2/3 (*L* = 194 mm), and the entire 3/3 (*L* = 300 mm) of the product duct. The $\bar{\mathrm{Nu}}$ in these calculations were derived using cumulatively averaged values of *h*_p_ starting from the die inlet and up to these nominal length thresholds, with the *L* term in the Gz^−1^ calculation (inverse of Equation 2) reflecting the same total die length for which the respective $\bar{\mathrm{Nu}}$ were averaged over.

## 3 Results and Discussion

### 3.1 Overall and Convective Heat Transfer Coefficient Results

The heat transfer coefficients (*U*_p_, *h*_m_, and *h*_p_) calculated for each discrete nominal 1/3, 2/3, and 3/3 of the die lengths used in this work, along with the *Ṁ*_p_, *Ṁ*_m_, and temperatures needed to calculate these values as per Equations (4)–(9), are documented in Table 4 (see the Supporting Information data for the *Ṁ*_p_, *Ṁ*_m_, *A*_p_, *A*_m_, and relevant temperatures needed to calculate these values; the thermophysical properties required are shown in Figure 2).

**TABLE 4:** Heat transfer coefficients, averaged over each nominal 1/3 of the cooling die's axial length.

			Heat transfer coefficients (W/m^2^∙k)																	
			a* = 0.45									a* = 0.28								
Data series	Ṁ_p_ (kg/hr)	*T*_m_ (°C)	Nominal 1/3			Nominal 2/3			Nominal 3/3			Nominal 1/3			Nominal 2/3			Nominal 3/3		
			*U*_p_	*h*_m_	*h*_p_	*U*_p_	*h*_m_	*h*_p_	*U*_p_	*h*_m_	*h*_p_	*U*_p_	*h*_m_	*h*_p_	*U*_p_	*h*_m_	*h*_p_	*U*_p_	*h*_m_	*h*_p_
**Original bookend points**	2.7	36.0	544, 446	699, 820	995, 653	163, 153	670, 786	187, 171	134, 133	662, 774	151, 147	603, 603	796, 783	1343, 1373	182, 183	749, 739	216, 219	166, 164	738, 729	197, 196
		48.0	502, 491	692, 794	867, 766	144, 128	670, 766	163, 141	115, 102	664, 759	128, 111	601, 629	769, 770	1396, 1550	193, 170	732, 734	233, 201	181, 147	723, 727	220, 171
		60.0	516, 533	725, 779	881, 886	140, 223	704, 754	156, 264	112, 222	699, 745	123, 267	632, 670	773, 769	1560, 1828	212, 202	742, 739	261, 246	212, 188	735, 733	266, 229
		72.0	592, 595	760, 750	1082, 1106	184, 186	741, 730	212, 215	157, 163	736, 725	178, 187	680, 674	775, 772	1879, 1844	263, 240	750, 747	342, 304	301, 255	744, 742	422, 336
	4.5	36.0	607, 608	713, 819	1205, 1069	202, 160	693, 794	239, 178	163, 122	687, 787	189, 133	779, 787	785, 792	2806, 2848	252, 167	749, 759	324, 195	219, 123	739, 753	277, 139
		48.0	635, 623	695, 804	1353, 1136	190, 136	678, 782	223, 149	148, 99	673, 778	168, 106	872, –	788, –	4469, –	214, –	757, –	262, –	168, –	751, –	199, –
		60.0	578, 694	708, 785	1102, 1430	109, 210	696, 766	119, 245	76, 172	694, 760	80, 197	815, 844	759, 779	3730, 3988	273, 176	735, 756	361, 208	242, 131	729, 752	316, 149
		72.0	564, 678	721, 761	1036, 1411	89, 93	711, 748	96, 100	60, 62	710, 746	63, 65	850, 830	771, 755	4283, 4159	259, 281	752, 735	335, 375	221, 254	747, 730	279, 337
New supplem‐entary points	2.7	54.0	496	775	789	258	745	316	263	735	329	538	778	1085	206	746	252	192	739	234
	2.96	41.3	451	798	672	247	752	298	171	755	196	529	802	1016	247	751	315	177	754	212
		66.7	476	774	741	200	764	232	238	745	290	613	776	1440	197	764	237	245	745	319
	3.6	36.0	655	803	1249	187	768	215	153	759	172	632	820	1436	236	747	297	143	772	164
		54.0	535	786	887	190	760	219	182	753	210	628	799	1467	180	780	213	195	762	239
		54.0	496	784	786	237	757	283	155	753	175	709	776	2115	224	769	276	195	740	239
		72.0	488	775	770	216	761	254	207	752	246	653	768	1710	224	746	280	209	742	261
	4.25	41.3	563	784	967	245	755	296	203	747	240	667	803	1685	242	765	306	195	760	239
		66.7	576	776	1015	289	757	362	258	750	319	749	788	2424	233	769	291	199	760	244
	4.50	54.0	659	785	1289	294	759	369	255	752	315	705	779	2058	243	752	308	203	746	251

*Note*: Most cells have two entries in the original bookend points section from part I, with each entry corresponding to a different process replicate. See Tables S2, S3, and S4 for details such as temperatures, flow rates, and heat exchange areas that went into these calculations.

Abbreviations: Ṁ_p_ = product mass flow rate target, *T*_m_ = cooling media set temperature, *U*_p_ = overall heat transfer coefficient, *h*_m_ = cooling media convective heat transfer coefficient, *h*_p_ = product side convective heat transfer coefficient.

Intuitively, heat transfer rates should be highest early in a duct where the thermal boundary layer is least developed (Kakaç et al. 2012), and the trends noted in Table 4 generally support this. As the temperature differential between product and media decreases and the thermal boundary layers formed at each heat transfer surface come closer to convergence at the nominal 2/3 die progression, and even more so over the whole length of the die, it would be expected that the heat transfer coefficients will truncate. The *h*_p_ values found for the first section of the die are notably high, especially for the a* = 0.28 die, which is likely a reflection of the extreme temperature differential the product encounters immediately upon entering the cooling die; indeed, the values of h_p_ in the first nominal 1/3 of the die can be up to a magnitude higher than values of *h*_p_ downstream in the die.

Some nonintuitive phenomena are observed in the Table 4 data as well. For example, increasing *Ṁ*_p_ results in a relative increase for any particular coefficient (as would be expected), but only for the first nominal 1/3 of the axial die length (which is unexpected); this could be due to the product melt being fluid for this first die section, while becoming a solid in the latter die sections (thereby mitigating any convection‐enhancing effect the extra product flow rate may induce). Additional notable behavior includes there being a positive relationship between *T*_m_ and *U*_p_ (or *h*_p_, similarly), which is counterintuitive since smaller ΔT values between product and cooling media would be expected to have the opposite effect; it is plausible that the higher *T*_m_ enhances heat transfer early in the die by keeping the melt in a liquid and thus convective state longer than is possible at lower values of *T*_m_.

From this data, it can be shown that 70–85% of the total product temperature change across the die occurs in this first section; as progress is made along the die, the values of *U*_p_ and *h*_p_ begin to converge as conduction becomes the prominent form of heat transfer on the product side. As inferred in this discussion so far, this would be consistent with much of the product cross section solidifying early in the die, which could also induce some degree of slip flow. To date, the studies that examine flow in the HMMA cooling die all agree that the product enters the die under a shear flow situation and then transitions to a plug (or slip) flow condition due to product solidification at some point in the die (Kaunisto et al. 2024; Sandoval Murillo et al. 2019; Wagner et al. 2024). While the exact timing of this transition is unknown given the current state of knowledge, the evidence presented here would suggest that rapid cooling in the die could cause solidification of the product surfaces close to the die wall so as to induce a partial slip flow very early in the cooling process. With so much heat exiting the product so early in the die, it would be logical in future work to alter the cooling media temperatures and flow rates in the die on a section‐by‐section basis to slow the heat transfer rate early in the die and thus perhaps better utilize more of the cooling die length. *To conclude, the importance of controlling the heat transfer rate early in the die cannot be understated*.

### 3.2 Heat Transfer Model and the Impact of the Effective Duct Length

**FIGURE 4:**
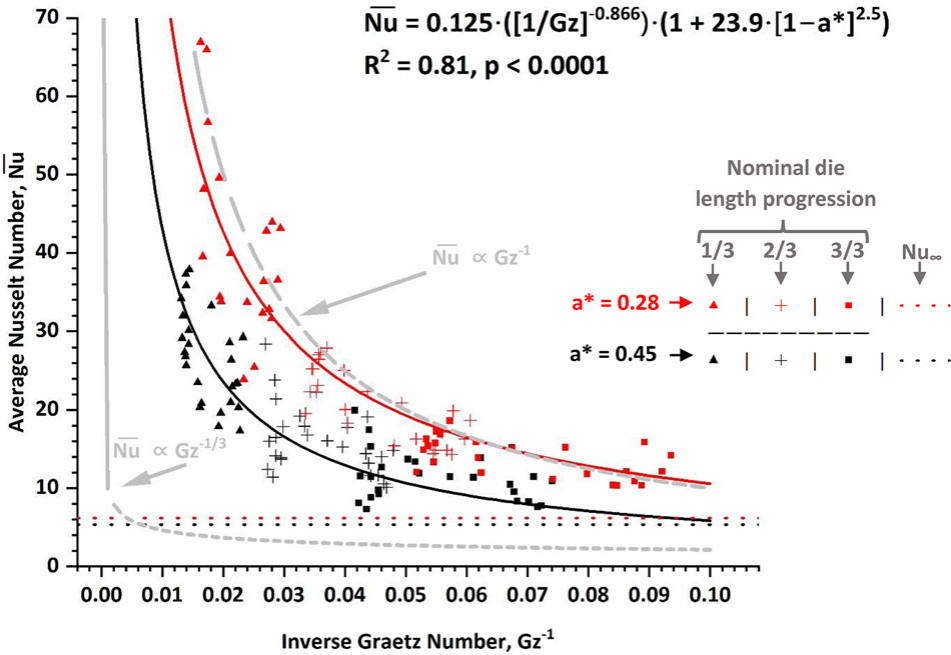
A dimensionless heat transfer model showing average Nusselt number ($\bar{\mathrm{Nu}}$) as a function of inverse Graetz number (Gz^−1^) and product conduit aspect ratio (a*). The model is provided in the upper right‐hand quadrant. The horizontal asymptotes show the theoretical fully developed Nusselt number (Nu_∞_) that would demarcate the theoretical full thermal development of the HMMA system. The gray boundaries correspond to the theoretical low Gz^−1^ asymptote ($\bar{\mathrm{Nu}}$ ∝ Gz^−1/3^) and theoretical high Gz^−1^ asymptote ($\bar{\mathrm{Nu}}$ ∝ Gz^−1^).

Figure 4 shows the $\bar{\mathrm{Nu}}$ = f(Gz^−1^, a*) model derived from the data. The fit is satisfactory for an empirical model (*R* ^2^ = 0.81, *p* < 0.0001). The parameters and their associated error (*α* = 0.05) are shown in Table 5 for reference. The predictive accuracy of the model, as inferred from the root mean square error provided in Table 5, is in the range of $\bar{\mathrm{Nu}}$ ± 4.85.

**TABLE 5:** Nonlinear parameter values obtained when fitting $\bar{\mathrm{Nu}}$, Gz^−1^, and a* values to Equation (10).

Equation (10) parameters	Parameter value	Parameter significance
A	0.125 ± 0.133	0.0649
B	−0.866 ± 0.076	<0.0001
C	1	N/A
D	23.9 ± 25.4	0.0646
E	2.5	N/A

*Note*: *R* ^2^ = 0.8088, adjusted *R* ^2^ = 0.8062, root mean square error = 4.853. The ± value indicates the 95% confidence interval of each fit value. Parameters *C* and *E* do not have precision or significance values since they were treated as fixed variables in the final fitting of Equation (10).

Since no similar HMMA heat transfer correlations exist in the literature for comparison, an examination of the model's asymptotic behavior was used to assess the reasonableness of fit parameter B. Proportions of $\bar{\mathrm{Nu}}$ ∝ Gz^−B^ must have values of B that range from −1/3 for low Gz^−1^ to −1 for high Gz^−1^ values, defined here as the asymptotic constant slope case for low Gz^−1^ values (i.e., very short heat exchanger length limiting case) and zero slope thermally fully developed case at high Gz^−1^ values (i.e., very long heat exchanger length limiting case), respectively (Bennett 2020; Carreau et al. 1997). The data in Figure 4 (black and red trends) manifest within these limits (gray trends) differently as progress through the die is made. Early in the die, where the flow is most thermally undeveloped (i.e., low Gz^−1^), a great deal of convection‐driven heat flux occurs, and the trends for either die look similar to the nearly vertical asymptote provided by $\bar{\mathrm{Nu}}$ ∝ Gz^−1/3^ (Figure 4). This potential for transferring heat diminishes greatly later in the die when the thermal boundary layers within the product begin to converge (i.e., high Gz^−1^ values). This long‐term high Gz^−1^ case should follow the $\bar{\mathrm{Nu}}$ ∝ Gz^−1^ boundary trend (Carreau et al. 1997), with the magnitude of $\bar{\mathrm{Nu}}$ not dropping below the theoretically fully developed $\bar{\mathrm{Nu}}$ (denoted Nu_∞_) case (Figure 4). The values of the Nu_∞_ asymptotes are geometrically dependent on the value of a*, and were calculated to be 6.19 and 5.35 for the respective a* = 0.28 and a* = 0.45 dies using data available from Shah & London (1978) (see Supporting Information data); the $\bar{\mathrm{Nu}}$ values obtained at larger values of Gz^−1^ for either die trend between these limits. To summarize, the power of *B* = −0.866 achieved for the Gz^−1^ term of Equation (10) is notable as it implies the thermal development of HMMA lies somewhere between these limits on average, with parallelism to a given limit being dictated by what die position is being assessed. A practical interpretation of this is that HMMA experiences both major forms of non‐radiative heat transfer within one realistically sized heat exchanger unit, from aggressive convection early in the die (when the product is in a liquid state) to mostly conduction late in the die (once the product has solidified). It is interesting to note that the a* = 0.28 trend follows the $\bar{\mathrm{Nu}}$ ∝ Gz^−1^ boundary trend very closely, save for at the very beginning of the duct (i.e., low Gz^−1^ numbers); this implies that the thermal boundary layers in the product being made in this die converged almost immediately upon entering the die, a likely reflection of the short die height and thus rapid equilibration of the temperature across the product crosssection at any given linear point in the die. It was argued in the study by Wagner et al. (2024) that the product made using the same a* = 0.28 die produced HMMA with relatively poor fibrousness due do the product cross section solidifying almost immediately, thus not allowing and kind of differential flow across the cross section to occur as would be necessary for fibrousness to develop; the adherence of the a* = 0.28 trend to the $\bar{\mathrm{Nu}}$ ∝ Gz^−1^ asymptote is a more quantitative expression of this theory.

The fit results of A, C, D, and E pertinent to the a* portion of the final model have less quantitative roots at this time. The value of *E* = 2.5 that was ultimately achieved does imply that parabolic behavior is close to being quadratic, which is reasonable since symmetry in a $\bar{\mathrm{Nu}}$ ∝ a* relationship would be expected given the relevant theoretical results for rectangular ducts presented in Shah & London (1978). Intuitively, a highly symmetrical relationship should be expected, as a given set of a* values that are inverses of each other (e.g., a* = 0.5 or a* = 2) should produce the exact same heat transfer rates assuming all sides of a rectangular duct offer the same resistance to heat transfer. What is less easily defined in the current work are the shape factors of this parabolic relationship, namely parameters C and D, with the former being encapsulated as A∙C as presented due to the factoring procedure discussed in Section 2.4. It is expected that the values of A, C, and D would all be better defined if data from a wider variety of rectangular geometries than just 2 were tested in future experimentation.

### 3.3 Comparison to Data from a Third Party

Data reconstructed from Lee et al. (2005) was used to test the model here since it is the only other publicly available HMMA‐relevant study to report the temperature and flow rate data required to the best of our knowledge.

While the results of this exercise are academically interesting, little practical information was gained. In their work, Lee et al. (2005) only reported overall heat transfer coefficients, with no meaningful dimensional analysis based on the data given as was done here. When extracting dimensionless terms from the dimensional data presented in Lee et al. (2005), it was found that those authors captured a Gz^−1^ = 0.001–0.004 range of values, which is very low compared with what was measured in this work. For this reason, the model from Figure 4, based on the work here, will not predict the Lee et al. (2005) data well due to extreme extrapolation of the Gz^−1^ values. In addition, it was speculated that nonpenetrative thermocouples were used in the Lee et al. (2005) work given the relatively low apparent $\bar{\mathrm{Nu}}$ calculated from their data, so the data in the Lee et al. (2005) paper may not be comparable with data where extensive thermocouple penetration into the product stream was performed like here (Mulvaney and Tsai 1996). To conclude, too much is unknown about the Lee et al. (2005) data to reliably be compared with the data here.

## 4 Conclusion

Heat transfer relationships were developed here as a function of the HMMA process cooling die geometry, flow rate, and thermal properties. The resulting models are well bounded within theoretical limits and illustrate quantitatively how the rate of heat transfer drops precipitously over the length of the cooling die due to product solidification. To the best of our knowledge, these are the first such models relevant to HMMA in the literature. They should be immediately useful for the food process engineer working with alternative meat analogs, as they provide reasonable estimates of the heat transfer coefficients for cooling die design purposes. This enables more accurate predictions of requirements when scaling up a HMMA cooling process, such as specifying the length of the cooling die, predicting the effect of changing product aspect ratio on heat transfer, defining how many product outlets may be needed to maintain a certain heat transfer rate, or determining the required cooling media flow rates and temperatures. Future work could be done to improve the structure and accuracy of the model by collecting data over a broader range of aspect ratios. A more thorough understanding of the material solidification temperatures, how to control them in the die, and how the onset timing of solidification affects heat transfer in a die could also lead to further refinements of the model.

## Nomenclature

**Constants, variables, and calculated values (Latin)**
A_c_
: conduit cross sectional area (m^2^)
C_p_
: heat capacity (kJ∙kg^‒1^∙K^‒1^)
$D_{h}\equiv \frac{4\cdot A}{P}$
: hydraulic or equivalent diameter (m)
H: conduit half height (m)
h: convective heat transfer coefficient (W∙m^‒2^∙K^‒1^)
k: thermal conductivity coefficient (W∙m^‒1^∙K^‒1^)
L: conduit length (m)
$\dot{M}$
: stream mass flow rate (kg∙s^‒1^)
P: conduit wetted perimeter (m)
T: temperature variable (K)
T® ≡ (T_i_+T_o_)/2: bulk average stream temperature (K)
$\Delta T_{ln}$
: log mean temperature (K)
T_wall_ ≡ (T®_p_ + T®_m_)/2: approximate wall temperature (K)
U: overall heat transfer coefficient (W∙m^‒2^∙K^‒1^)
V®
: average stream velocity (m∙s^‒1^)
W: conduit half width (m)

**Constants, variables, and calculated values (Greek)**
ρ: density (kg∙m^‒3^)
μ: viscosity coefficient (Pa∙s)

**Variables with space dependencies**
V_x_ (Y,Z): V_x_, axial velocity component (m∙s^‒1^)
T(X,Y,Z): T, local fluid temperature (K)
T®(X): T®, bulk average product temperature corresponding to a cross‐sectional slice dX (K)
T_0_ (X): T_0_, average interface temperature corresponding to a cross‐sectional slice dX (K)
X: axial coordinate (m)
Y: vertical coordinate (m)
Z: horizontal coordinate (m)

**Dimensionless variables**
⌣X
: X/L
⌣Y
: Y/Dh
Ž: Z/Dh
Θ
: $\;\frac{T\;-T_{0}}{T_{b}-T_{0}}$
a*: 2H/2W
$\mathrm{Gz}\;\equiv \frac{\rho \cdot C_{p}\cdot \bar{V}\cdot {D_{h}}^{2}}{L\cdot k}$
: Graetz number
$\mathrm{Nu}\;\equiv \;\;\frac{h\cdot D_{h}}{k}$
: Nusselt number
Re≡ρ·V¯·Dhμ
: Reynolds number
$\mathrm{Pr}\;\equiv \frac{\mu \cdot C_{p}}{k}$
: Prandtl number

**Subscripts**
m: defined for the cooling media or media conduit
p: defined for the product or product conduit
i: inlet
o: outlet
X: axially local value
wall: defined for the heat exchanger wall

## Author Contributions

Corresponding author **Girish M. Ganjyal** was responsible for project conceptualization, funding acquisition, and review of the manuscript. First author Caleb Wagner designed the experiments, collected the data, analyzed the data, and drafted the manuscript. The second author, **Leon Levine**, conceptualized the dimensional analysis, helped analyze the data, and reviewed the manuscript extensively.

## Conflicts of Interest

The authors declare no conflicts of interest.

## Supporting information




**Supplementary material**: jfds70366‐sup‐0001‐SuppMat.docx
