# Supplementary material for: A Dimensionless Empirical Model to Predict Heat Transfer Coefficients for Cooling High‐Moisture Meat Analog with Rectangular Dies
Source: J Food Sci. 2025 Jul 5;90(7):e70366. doi: 10.1111/1750-3841.70366 (PMC12228055; doi:10.1111/1750-3841.70366)
Supplement: Supplementary file 1 — Supplementary material: jfds70366‐sup‐0001‐SuppMat.docx [file JFDS-90-0-s001.docx]

**Dimensional scaling of the heat equation**

Figure 1 of the main text shows a diagram of the system here, with corresponding boundary conditions being listed in Table 2. The system can be described as a laminar flow cooling under steady state conditions in a rectangular duct. For brevity in text, quantities that depend on position (e.g., T0 [X], T̅[X], T[X,Y], VX[Y,Z]) will be referred to without their position-function declaration (i.e., just T0, T̅, T, and Vx, respectively).

The full transient heat equation with constant density, heat capacity, and conduction proportion constants is expressed as follows (Bird et al., 2007; Geankoplis et al., 2018)

$\frac{\text{∂T}}{\text{∂t}}\text{+ ρ}\text{ C}_{\text{p}}\text{ (}\text{V}\text{∙}\text{∇}\text{T) = k }\text{∇}^{\text{2}}\text{T + μ }\text{∇}^{\text{2}}\text{V}+\dot{q}$ (S1)

Applying the assumptions in Table 2 results in the following differential equation.

$\text{ρ}\text{ C}_{\text{p}}\text{ V}_{\text{X}}(Y,Z)\frac{\text{∂T}}{\text{∂X}}\text{=k (}\frac{\text{∂}^{\text{2}}\text{T}}{\text{∂}\text{Y}^{\text{2}}}$+ $\frac{\text{∂}^{\text{2}}\text{T}}{\text{∂}\text{Z}^{\text{2}}}$) (S2)

Which states that the heat advected axially (X) into the cooling die product duct with the product flow is balanced by conduction at the duct periphery (Y and Z directions). An explanation of the assumption in Table 3, besides the generally accepted 1-3 for a noncompressible steady flow with no accumulation, are as follows: Negligible viscous dissipation of heat in the melt (assumption 4), which can be proven to be negligible for this system using frictional pressure drop calculations (Wagner et al., 2024); Vertical and horizontal velocity components can be neglected (assumption 5) since there is no net product flow in those directions; Quantity of heat being conducted axially (assumption 6) is very small compared to the quantity of heat being advected in the same direction; Heat generation during the process (assumption 7), specifically latent energy release during cooling and frictional heating, is small compared to the sensible heat exchange quantity (Wagner et al., 2024).

A dimensionless temperature (Θ) can be developed in consideration of the dimensional boundary conditions in Table 1.

$\Theta=\frac{T_{p,xyz}-T_{m}}{T_{p,i}-T_{m}}$ (S3)

Where product T_p,xyz_ is product temperature as a function of position in the duct, T_m_ is the cooling media temperature, and T_p,i_ is the product inlet temperature. Taking the 1^st^ and 2^nd^ derivative of equation S3 with respect to T_p,xyz_ and rearraning:

$\text{∂}\text{T}_{\text{p,xyz}}$ $\text{=∂}\text{Θ}\text{ (T}_{\text{p,i}}\text{-}\text{T}_{\text{m}}\text{)}$ (S3’)

$\text{∂}^{\text{2}}\text{T}_{\text{p,xyz}}$ $\text{=}\text{∂}^{\text{2}}\text{Θ}\text{ (T}_{\text{p,i}}\text{-}\text{T}_{\text{m}}\text{)}$ (S3’’)

The system dimensions X, Y, and Z can be can be rendered dimensionless by utilizing L and D_h_ as suggested by Suzzi & Lorenzini (2019):

$\text{X̌ = }\frac{X}{\text{L}}$ (S4)

$\text{Y̌}\text{ = }\frac{Y}{D_{h}}$ (S5)

$\text{Ž}\text{ = }\frac{Z}{D_{h}}$ (S6)

Where L is the channel length and D_h_ is the hydraulic (or equivalent) diameter. It was chosen to use D_h_ as the scaling varible for the Y and Z dimensions to help account fo the finite slit width. These variables can be derivatized similial to equation S3’ and S3’’.

$\text{∂X }\text{=}\text{ L ∂X̌ }$ (S4’)

$\partial Y= D_{h} \partial\text{Y̌}$ (S5’)

$\partial Y^{2} = {D_{h}}^{2}\text{Y̌}^{2}$ (S5’’)

$\partial Z = D_{h} \partial\text{Ž}$ (S6’)

$\partial Z^{2} = {D_{h}}^{2}\partial\text{Ž}^{2}$ (S6’’)

A generalized velocity profile for HMMA is difficult to describe quantitatively since the product enters he die as a non-slipping liquid and then transitions to a slip-flow solid upon discharge. However, taking advantage of the fact that *any* velocity profile for a constant volumetric flow situation can be expressed as a function of the conduit geometry and average cross sectional velocity, we can express the V_x_(Y,Z) term in Equation S2 for an arbitrary rectangular cross section as

$\frac{\text{V}_{\text{X}}\left( Y,Z \right)}{\bar{V}}=f(X,Y,Z,\frac{H}{W})$ (S7)

Where H and W are the conduit half height and width, respectively. It can be shown that the H and W parameters appear when expressing the Hagen-Poiseulle law for a rectangular cross section as a function of these geometric parameters and V_avg_. As such, by logic,

$\frac{\text{V}_{\text{X}}\left( \text{Y,Z} \right)}{\bar{\text{V}}}\text{=f(}\check{\text{Z}}\text{,}\check{\text{Y}}\text{, }\text{X̌}\text{,}\frac{\text{H}}{\text{W}}\text{)}$ (S8)

Substituting all Equations shown from S3’–S8 into Equation S2 and simplifying appropriately results results in a dimensionless form of the differential equation.

$\text{Gz }f\left( \check{\text{X}},\check{\text{Y}}, \check{\text{Z}},\frac{H}{W} \right)\frac{\text{∂}\text{Θ}}{\text{∂}\text{X̌}}\text{=}\frac{\text{∂}^{\text{2}}\text{Θ}}{\text{∂}\text{Y̌}^{\text{2}}}+\frac{\text{∂}^{\text{2}}\text{Θ}}{\text{∂}{Ž}^{\text{2}}}$ (S9)

Where the differential terms $\frac{\text{∂}\text{Θ}}{\text{∂}\text{X̌}}$ , $\frac{\text{∂}^{\text{2}}\text{Θ}}{\text{∂}\text{Y̌}^{\text{2}}},$ and $\frac{\text{∂}^{\text{2}}\text{Θ}}{\text{∂}{Ž}^{\text{2}}}$ are the dimensionless axial heat advection, vertical conduction, and horizontal conduction terms, respectively. It is important to note the dimensionless Graetz number (Gz) that appeared during the procedure.

$\text{Gz ≡ }\frac{\text{ρ}\text{ }\text{C}_{\text{p}}\text{ }\bar{\text{V}}\text{ }{\text{D}_{\text{h}}}^{\text{2}}}{\text{L k}}$ (S10)

Since the boundary values of the dimensionally scaled equation all range from 0–1 as per table 2, and knowing that the solution to a differential equation must be a function of the variables and parameters that appear in the original equation, then by logic

$\text{Θ}\text{ = g(}\check{\text{X}}\text{,}\check{\text{Y}}\text{, }\check{\text{Z}}\text{, }\frac{\text{H}}{\text{W}}\text{, Gz)}$ (S11)

The conduction rate can be modeled as a function of the local convection rate at the heat exchange surface (Bird et al., 2007; Valentas et al., 1991). It is understood here that the media conduits only interface with the X–Z planes (i.e., top and bottom of the faces of the product conduit as per Figure 1), and thus this balance is directly relevant to the vertical (Y) direction temperature gradient.

$\text{-}\text{k }\frac{\text{∂T}}{\text{∂Y}}\text{|}_{\text{Y=±H}}\text{=}\text{h}_{\text{X}}\text{ (}\bar{\text{T}}\text{-}\text{T}_{\text{0}}\text{)}$ (S12)

Where $\bar{\text{T}}$ is the bulk average product temperature across any given Y–Z crossection and T_0_ is the wall temperature corresponding to a differential slice of the product conduit and heat transfer boundary as shown in Figure 1. The $\bar{\text{T}}$ is used here since it is easier to measure a bulk average product temperature rather than an interface temperature, and can be defined explicitly here as a mass average in terms of dimensionless positions as:

$\bar{\text{T}}\left( \check{\text{X}} \right)\text{=}\frac{\text{1}}{\dot{\text{M}}}\iint_{\text{0,0}}^{\text{1,1}} \text{ }\dot{\text{V}}\text{(}\check{\text{X}}\text{,}\check{\text{Y}}\text{,}\text{ }\check{\text{Z}}\text{)∙T(}\check{\text{X}}\text{,}\check{\text{Y}}\text{,}\text{ }\check{\text{Z}}\text{)}\text{ }$d$\check{\text{Y}}$ d$\check{\text{Z}}$ (S13)

Where Ṁ and V̇ are the product mass and volumetric flow rates, respectively, and are assumed equal since product density is constant. The use of the local heat transfer coefficient (h_x_) multiplied by the difference $\bar{\text{T}}$-T_0_ is detailed by Bird et al. (2007), and is the key simplification required to translate experimental temperature measurements into empirical correlation data. Applying the same dimensionless parameters utilized to change Equation 2 in to the dimensionless Equation 9 and simplfying results in the appearance of the local Nusselt number, (Nu_X_) as a function of dimensionless temperature vertical gradient evaluated between the two die dimensionless wall boundaries.

$\text{Nu}_{\text{X}}\text{= }\frac{\text{1}}{\bar{\text{T}}}\text{ }\frac{\text{∂}\text{Θ}}{\text{∂Y̌}}\text{|}_{\text{Y̌=±}\frac{\text{H}}{\text{D}_{\text{h}}}}$ (S14)

Where the Nusselt number (Nu) is defined as follows.

$\text{Nu }\text{≡ }\text{ }\frac{\text{h }\text{D}_{\text{h}}}{\text{k}}$ (S15)

Since the dimensionless temperature ($\text{Θ)}$ is defined as per Equation S11, it follows that the dimensionless vertical temperature gradient ($\frac{\text{∂}\text{Θ}}{\text{∂Y̌}}$) in equation S13 will be a function of the same variables and parameters. Evaluating at the $\text{Y̌=±}\frac{\text{H}}{\text{D}_{\text{h}}},$ averaging across the width ($\check{\text{Z}})$, and acknowledging that we defined T̄ as a function of $\check{\text{X}}$in Equation S13, we find that

$\text{Nu}_{\text{X}}\text{= f(Gz,}\frac{\text{H}}{\text{W}}\text{, }\text{X̌)}$ (S16)

The average Nusselt number (N͞u) can be found by averaging the Nu_X_ values over the length of the die, eliminating $X̌$ as a variable.

$\bar{\text{Nu}}\text{= }\int_{\text{X̌=}\text{0}}^{\text{X̌=}\text{1}} \text{f(Gz,}\frac{\text{H}}{\text{W}}\text{, }\text{X̌)}\text{d}\text{X̌ = f(}\text{Gz,}\frac{\text{H}}{\text{W}}\text{)}$ (S17)

Defining the aspect ratio as H/W = a* for convenience, we find a useful correlation for the purposes of this work takes the form of Equation S18.

$\bar{\text{Nu}}\text{ = }$f(Gz, a*) (S18)

It is notable that this dimensional scaling process utilized an arbitrary velocity profile, which is a necessary formality given the rheology of this system is not well defined and that the cooling product experiences various degrees of slip as stated. The key point of this exercise is that the solution will always collapse to a function of Gz and a* for a rectangular duct, even when a more explicit velocity profile is utilized. If the rheology of the product while still in a liquid flowable state were better defined, it is anticipated that it would be beneficial to incorporate power-fluid behavior into the model. While not detailed here, it can be shown that this exercise would result in the same correlation as Equation 2 but with an additional flow index (n) variable.


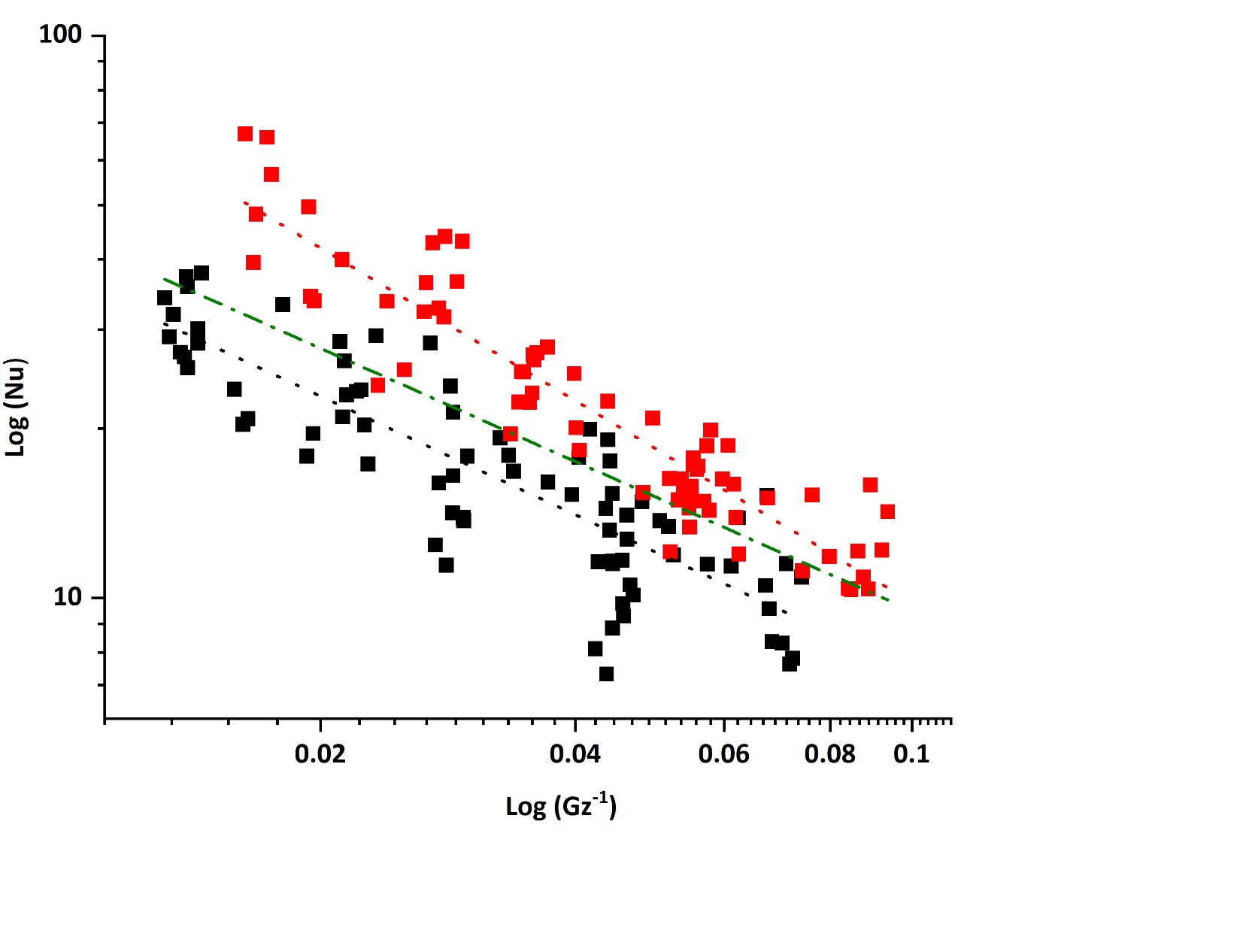


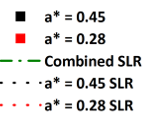


| **Specified partition** | **Slope** | **Intercept** | **R^2^** | **p-value** |
| --- | --- | --- | --- | --- |
| **None** | -0.668 | 0.309 | 0.56 | <0.0001 |
| **By a* = 0.45** | -0.699 | 0.171 | 0.73 | <0.0001 |
| **By a* = 0.28** | -0.902 | 0.090 | 0.86 | <0.0001 |

**Figure S1** The simple linear regressions (SLR) fit to logarithmically linearized N͞u and Gz^-1^used as part of the model justification process. Note, the clear partition between die types here serves to justify the use of the power law model used.

**Table S1** Inscribed central composite design used to supplement bookend factorial data fromWagner et. al., 2024.

| **Experiment** | **Die temperature, °C (X1)** | **Ṁ_p_, kg/hr (X2)** | **X1, coded** | **X2, coded** |
| --- | --- | --- | --- | --- |
| 1 | 41.27 | 2.96 | -0.71 | -0.71 |
| 2 | 66.73 | 2.96 | 0.71 | -0.71 |
| 3 | 41.27 | 4.24 | -0.71 | 0.71 |
| 4 | 66.73 | 4.24 | 0.71 | 0.71 |
| 5 | 54.00 | 3.60 | 0.00 | 0.00 |
| 6 | 36.00 | 3.60 | -1.00 | 0.00 |
| 7 | 72.00 | 3.60 | 1.00 | 0.00 |
| 8 | 54.00 | 2.70 | 0.00 | -1.00 |
| 9 | 54.00 | 4.50 | 0.00 | 1.00 |
| 10 | 54.00 | 3.60 | 0.00 | 0.00 |

**Table S2** Locally averaged heat transfer coefficients and corresponding data needed for their calculation for the nominal 1^st^ 1/3 of the die. Note the data for the locally averaged and cumulatively averaged values are the same for this particular die section.

|  |  | |  | a* = 0.45 | | | | | | | | | | |  | a* = 0.28 | | | | | | | | | | |
| --- | --- | --- | --- | --- | --- | --- | --- | --- | --- | --- | --- | --- | --- | --- | --- | --- | --- | --- | --- | --- | --- | --- | --- | --- | --- | --- |
| Experimental series  and process targets | | |  | Measured mass flow rate (kg/hr) | |  | Temperature  (°C) | | | |  | Heat transfer coefficients  (W/m^2^∙k) | | |  | Mass flow rate (kg/hr) | |  | Temperature  (°C) | | | |  | Heat transfer coefficients  (W/m^2^∙k) | | |
| Series | Ṁ_p_  (kg/hr) | T_m_  (°C) |  | Ṁ_p_ | Ṁ_m_ |  | T_m,i_ | T_m,o_ | T_p,i_ | T_p,o_ |  | U_p_ | h_m_ | h_p_ |  | Ṁ_p_ | Ṁ_m_ |  | T_m,i_ | T_m,o_ | T_p,i_ | T_p,o_ |  | U_p_ | h_m_ | h_p_ |
| Original bookend points | 2.7 | 36.0 |  | 2.85, 2.67 | 23.6, 37.2 |  | 38.4, 37.3 | 43.9, 41.1 | 150.2, 149.5 | 87.4, 91.5 |  | 544, 446 | 699, 820 | 995, 653 |  | 2.71, 2.86 | 35.5, 33.8 |  | 37.6, 38.0 | 41.7, 42.1 | 148.6, 148.3 | 70.6, 73.0 |  | 603, 603 | 796, 783 | 1343, 1373 |
|  |  | 48.0 |  | 2.83, 2.70 | 23.9, 36.0 |  | 49.8, 48.8 | 54.4, 52.4 | 149.5, 147.9 | 96.1, 93.7 |  | 502, 491 | 692, 794 | 867, 766 |  | 2.75, 2.85 | 33.5, 33.7 |  | 49.1, 49.4 | 52.9, 53.2 | 148.3, 148.2 | 79.3, 79.1 |  | 601, 629 | 769, 770 | 1396, 1550 |
|  |  | 60.0 |  | 2.76, 2.72 | 28.4, 35.4 |  | 60.6, 60.1 | 64.5, 63.5 | 148.6, 147.3 | 99.8, 97.4 |  | 516, 533 | 725, 779 | 881, 886 |  | 2.62, 2.80 | 35.3, 34.8 |  | 60.2, 60.7 | 63.6, 64 | 147.2, 147.5 | 83.7, 84.4 |  | 632, 670 | 773, 769 | 1560, 1828 |
|  |  | 72.0 |  | 2.89, 2.61 | 33.7, 32.5 |  | 71.9, 71.4 | 74.2, 74.5 | 147.9, 146.2 | 103, 99.3 |  | 592, 595 | 760, 750 | 1082, 1106 |  | 2.71, 2.59 | 36.3, 35.9 |  | 71.5, 72.0 | 74.6, 74.8 | 147.1, 147.6 | 90.9, 90.4 |  | 680, 674 | 775, 772 | 1879, 1844 |
|  | 4.5 | 36.0 |  | 4.54, 4.24 | 25.0, 37.7 |  | 39.5, 38.5 | 45.5, 42.3 | 142.6, 142.9 | 97.5, 94.4 |  | 607, 608 | 713, 819 | 1205, 1069 |  | 4.39, 4.66 | 34.3, 35.0 |  | 39.4, 39.1 | 44.3, 43.8 | 143.8, 143.9 | 79.3, 81.0 |  | 779, 787 | 785, 792 | 2806, 2848 |
|  |  | 48.0 |  | 4.37, 4.24 | 24.4, 37.4 |  | 50.9, 49.5 | 56.3, 53.5 | 143.9, 144.1 | 100.7, 99.5 |  | 635, 623 | 695, 804 | 1353, 1136 |  | 4.50, – | 36.4, – |  | 50.0, – | 54.4, – | 143.5, – | 82.8, – |  | 872, – | 788, – | 4469, – |
|  |  | 60.0 |  | 4.59, 4.20 | 26.5, 36.4 |  | 61.6, 61.4 | 65.8, 64.8 | 142.3, 142.5 | 108.5, 101.1 |  | 578, 694 | 708, 785 | 1102, 1430 |  | 4.38, 4.43 | 33.4, 36.1 |  | 61.4, 61.6 | 65.4, 65.3 | 142.7, 143.5 | 91.2, 90.8 |  | 815, 844 | 759, 779 | 3730, 3988 |
|  |  | 72.0 |  | 4.46, 4.38 | 28.6, 33.8 |  | 72.8, 72.6 | 75.7, 75.7 | 142.8, 142.2 | 113.3, 108.3 |  | 564, 678 | 721, 761 | 1036, 1411 |  | 4.40, 4.23 | 35.8, 33.5 |  | 72.6, 72.9 | 75.9, 76.4 | 142.7, 144.3 | 97.3, 97.7 |  | 850, 830 | 771, 755 | 4283, 4159 |
| New Supplem-entary points | 2.7 | 54.0 |  | 2.86 | 34.9 |  | 54.4 | 57.4 | 144.9 | 86.8 |  | 496 | 775 | 789 |  | 3.02 | 35.5 |  | 55.0 | 58.4 | 144.7 | 83.4 |  | 538 | 778 | 1085 |
|  | 2.96 | 41.3 |  | 3.15 | 36.2 |  | 42.6 | 46.0 | 143.9 | 85.9 |  | 451 | 798 | 672 |  | 3.24 | 37.3 |  | 43.1 | 46.8 | 144.5 | 78.4 |  | 529 | 802 | 1016 |
|  |  | 66.7 |  | 3.10 | 35.4 |  | 66.9 | 69.5 | 145.5 | 98.6 |  | 476 | 774 | 741 |  | 3.17 | 36.3 |  | 67.1 | 70.1 | 144.4 | 89.3 |  | 613 | 776 | 1440 |
|  | 3.6 | 36.0 |  | 3.36 | 35.9 |  | 38.1 | 42.0 | 144.4 | 83.5 |  | 655 | 803 | 1249 |  | 3.86 | 38.9 |  | 38.3 | 42.5 | 145.5 | 75.5 |  | 632 | 820 | 1436 |
|  |  | 54.0 |  | 3.84 | 35.9 |  | 54.6 | 58.3 | 145.2 | 94.4 |  | 535 | 786 | 887 |  | 3.90 | 38.3 |  | 55.5 | 59.1 | 144.5 | 87.0 |  | 628 | 799 | 1467 |
|  |  | 54.0 |  | 3.70 | 35.6 |  | 54.8 | 58.3 | 145.1 | 95.7 |  | 496 | 784 | 786 |  | 3.92 | 35.3 |  | 55.6 | 59.6 | 146.1 | 83.9 |  | 709 | 776 | 2115 |
|  |  | 72.0 |  | 3.76 | 35.6 |  | 72.4 | 74.9 | 144.9 | 106.1 |  | 488 | 775 | 770 |  | 3.59 | 35.3 |  | 72.6 | 75.8 | 144.8 | 95.1 |  | 653 | 768 | 1710 |
|  | 4.25 | 41.3 |  | 4.36 | 34.3 |  | 43.3 | 47.9 | 144.0 | 90.4 |  | 563 | 784 | 967 |  | 4.56 | 37.2 |  | 43.9 | 48.0 | 145.4 | 83.4 |  | 667 | 803 | 1685 |
|  |  | 66.7 |  | 4.40 | 35.5 |  | 67.5 | 70.5 | 145.5 | 103.6 |  | 576 | 776 | 1015 |  | 4.35 | 37.8 |  | 67.8 | 71.1 | 144.4 | 93.1 |  | 749 | 788 | 2424 |
|  | 4.50 | 54.0 |  | 4.64 | 35.9 |  | 55.2 | 59.2 | 145.9 | 94.5 |  | 659 | 785 | 1289 |  | 4.53 | 35.6 |  | 56.0 | 60.3 | 144.9 | 88.8 |  | 705 | 779 | 2058 |

A_m_ = 6460 mm^2^ for all calculations. A_p_ = 3760 mm^2^ or A_p_ = 4700 mm^2^ for the a* = 0.45 or a* = 0.28 die, respectively. In the original bookend points section, most cells have two entries, with each entry corresponding to a different process replicate; unfortunately, the data for the 2^nd^ process replicate of the a* = 0.28, Ṁ_p_ = 4.5 kg/hr, and T_m_= 48 °C conditions was lost due to corruption of the data during file transfer.

**Table S3** Locally averaged heat transfer coefficients and corresponding data needed for their calculation for the nominal 2^nd^ 1/3 of the die.

|  |  | |  | a* = 0.45 | | | | | | | | | | |  | a* = 0.28 | | | | | | | | | | |
| --- | --- | --- | --- | --- | --- | --- | --- | --- | --- | --- | --- | --- | --- | --- | --- | --- | --- | --- | --- | --- | --- | --- | --- | --- | --- | --- |
| Experimental series  and process targets | | |  | Measured mass flow rate (kg/hr) | |  | Temperature  (°C) | | | |  | Heat transfer coefficients  (W/m^2^∙k) | | |  | Mass flow rate (kg/hr) | |  | Temperature  (°C) | | | |  | Heat transfer coefficients  (W/m^2^∙k) | | |
| Series | Ṁ_p_  (kg/hr) | T_m_  (°C) |  | Ṁ_p_ | Ṁ_m_ |  | T_m,i_ | T_m,o_ | T_p,i_ | T_p,o_ |  | U_p_ | h_m_ | h_p_ |  | Ṁ_p_ | Ṁ_m_ |  | T_m,i_ | T_m,o_ | T_p,i_ | T_p,o_ |  | U_p_ | h_m_ | h_p_ |
| Original bookend points | 2.7 | 36.0 |  | 2.85, 2.67 | 23.6, 37.2 |  | 36.4, 36.2 | 38.7, 37.4 | 85.0, 89.0 | 75.2, 78.1 |  | 163, 153 | 670, 786 | 187, 171 |  | 2.71, 2.86 | 35.5, 33.8 |  | 36.2, 36.3 | 37.7, 38.1 | 68.4, 70.7 | 59.2, 61.3 |  | 182, 183 | 749, 739 | 216, 219 |
|  |  | 48.0 |  | 2.83, 2.70 | 23.9, 36.0 |  | 47.8, 47.7 | 50, 48.6 | 94.1, 91.9 | 85.7, 84.2 |  | 144, 128 | 670, 766 | 163, 141 |  | 2.75, 2.85 | 33.5, 33.7 |  | 47.7, 47.8 | 49.0, 49.2 | 77.2, 77.3 | 68.5, 69.7 |  | 193, 170 | 732, 734 | 233, 201 |
|  |  | 60.0 |  | 2.76, 2.72 | 28.4, 35.4 |  | 59.2, 59.1 | 60.5, 60.1 | 98.1, 95.1 | 91.0, 85.0 |  | 140, 223 | 704, 754 | 156, 264 |  | 2.62, 2.80 | 35.3, 34.8 |  | 59.1, 59.4 | 60.2, 60.5 | 81.9, 82.7 | 74.4, 75.7 |  | 212, 202 | 742, 739 | 261, 246 |
|  |  | 72.0 |  | 2.89, 2.61 | 33.7, 32.5 |  | 70.6, 70.8 | 71.7, 71.6 | 101.4, 97.7 | 94.5, 91.1 |  | 184, 186 | 741, 730 | 212, 215 |  | 2.71, 2.59 | 36.3, 35.9 |  | 70.8, 71.1 | 71.3, 71.7 | 89.3, 88.8 | 82.2, 82.2 |  | 263, 240 | 750, 747 | 342, 304 |
|  | 4.5 | 36.0 |  | 4.54, 4.24 | 25.0, 37.7 |  | 37.0, 36.8 | 39.7, 38.5 | 95.3, 92.6 | 85.9, 84.8 |  | 202, 160 | 693, 794 | 239, 178 |  | 4.39, 4.66 | 34.3, 35.0 |  | 37.2, 36.9 | 39.4, 39.3 | 77.0, 79.4 | 67.1, 72.5 |  | 252, 167 | 749, 759 | 324, 195 |
|  |  | 48.0 |  | 4.37, 4.24 | 24.4, 37.4 |  | 48.4, 48.1 | 51.2, 49.7 | 98.9, 98.1 | 90.9, 92.1 |  | 190, 136 | 678, 782 | 223, 149 |  | 4.50, – | 36.4, – |  | 48.3, – | 50.0,– | 81.1, – | 74.2, – |  | 214, – | 757, – | 262, – |
|  |  | 60.0 |  | 4.59, 4.20 | 26.5, 36.4 |  | 59.8, 59.6 | 61.2, 60.9 | 107.5, 99.4 | 103.2, 92.2 |  | 109, 210 | 696, 766 | 119, 245 |  | 4.38, 4.43 | 33.4, 36.1 |  | 59.7, 59.9 | 61.4, 61.3 | 89.4, 89.5 | 81.5, 84.3 |  | 273, 176 | 735, 756 | 361, 208 |
|  |  | 72.0 |  | 4.46, 4.38 | 28.6, 33.8 |  | 71.3, 71.5 | 72.7, 72.4 | 112.5, 107.5 | 109.4, 104.6 |  | 89, 93 | 711, 748 | 96, 100 |  | 4.40, 4.23 | 35.8, 33.5 |  | 71.3, 71.5 | 72.4, 72.6 | 95.8, 96.1 | 89.6, 89.2 |  | 259, 281 | 752, 735 | 335, 375 |
| New Supplem-entary points | 2.7 | 54.0 |  | 2.86 | 34.9 |  | 53.4 | 54.4 | 84.6 | 75.1 |  | 258 | 745 | 316 |  | 3.02 | 35.5 |  | 53.7 | 54.9 | 81.5 | 73.5 |  | 206 | 746 | 252 |
|  | 2.96 | 41.3 |  | 3.15 | 36.2 |  | 65.8 | 66.9 | 96.6 | 88.3 |  | 247 | 752 | 298 |  | 3.24 | 37.3 |  | 66.0 | 67.0 | 87.7 | 80.7 |  | 247 | 751 | 315 |
|  |  | 66.7 |  | 3.10 | 35.4 |  | 41.2 | 43.7 | 83.7 | 74.3 |  | 200 | 764 | 232 |  | 3.17 | 36.3 |  | 41.5 | 43.0 | 76.2 | 67.1 |  | 197 | 764 | 237 |
|  | 3.6 | 36.0 |  | 3.36 | 35.9 |  | 36.6 | 38.2 | 81.4 | 72.5 |  | 187 | 768 | 215 |  | 3.86 | 38.9 |  | 71.4 | 72.6 | 93.6 | 87.5 |  | 236 | 747 | 297 |
|  |  | 54.0 |  | 3.84 | 35.9 |  | 53.5 | 55.1 | 93.9 | 86.5 |  | 190 | 760 | 219 |  | 3.90 | 38.3 |  | 36.4 | 38.3 | 73.7 | 65.9 |  | 180 | 780 | 213 |
|  |  | 54.0 |  | 3.70 | 35.6 |  | 71.3 | 72.3 | 104.4 | 97.1 |  | 237 | 757 | 283 |  | 3.92 | 35.3 |  | 53.7 | 55.4 | 85.2 | 77.4 |  | 224 | 769 | 276 |
|  |  | 72.0 |  | 3.76 | 35.6 |  | 53.5 | 54.7 | 92.6 | 84.7 |  | 216 | 761 | 254 |  | 3.59 | 35.3 |  | 54.1 | 54.1 | 82.2 | 75.1 |  | 224 | 746 | 280 |
|  | 4.25 | 41.3 |  | 4.36 | 34.3 |  | 41.4 | 44.1 | 88.2 | 78.9 |  | 245 | 755 | 296 |  | 4.56 | 37.2 |  | 66.4 | 67.9 | 91.7 | 85.6 |  | 242 | 765 | 306 |
|  |  | 66.7 |  | 4.40 | 35.5 |  | 66.3 | 67.5 | 101.7 | 93.6 |  | 289 | 757 | 362 |  | 4.35 | 37.8 |  | 41.9 | 43.8 | 81.3 | 72.5 |  | 233 | 769 | 291 |
|  | 4.50 | 54.0 |  | 4.64 | 35.9 |  | 53.8 | 55.1 | 92.5 | 83.9 |  | 294 | 759 | 369 |  | 4.53 | 35.6 |  | 54.2 | 56.0 | 87.0 | 79.4 |  | 243 | 752 | 308 |

A_m_ = 6460 mm^2^ for all calculations. A_p_ = 3400 mm^2^ or A_p_ = 4250  mm^2^ for the a* = 0.45 or a* = 0.28 die, respectively. In the original bookend points section, most cells have two entries, with each entry corresponding to a different process replicate; unfortunately, the data for the 2^nd^ process replicate of the a* = 0.28, Ṁ_p_ = 4.5 kg/hr, and T_m_= 48 °C conditions was lost due to corruption of the data during file transfer.

**Table S4** Locally averaged heat transfer coefficients and corresponding data needed for their calculation for the nominal last 1/3 of the die.

|  |  | |  | a* = 0.45 | | | | | | | | | | |  | a* = 0.28 | | | | | | | | | | |
| --- | --- | --- | --- | --- | --- | --- | --- | --- | --- | --- | --- | --- | --- | --- | --- | --- | --- | --- | --- | --- | --- | --- | --- | --- | --- | --- |
| Experimental series  and process targets | | |  | Measured mass flow rate (kg/hr) | |  | Temperature  (°C) | | | |  | Heat transfer coefficients  (W/m^2^∙k) | | |  | Mass flow rate (kg/hr) | |  | Temperature  (°C) | | | |  | Heat transfer coefficients  (W/m^2^∙k) | | |
| Series | Ṁ_p_  (kg/hr) | T_m_  (°C) |  | Ṁ_p_ | Ṁ_m_ |  | T_m,i_ | T_m,o_ | T_p,i_ | T_p,o_ |  | U_p_ | h_m_ | h_p_ |  | Ṁ_p_ | Ṁ_m_ |  | T_m,i_ | T_m,o_ | T_p,i_ | T_p,o_ |  | U_p_ | h_m_ | h_p_ |
| Original bookend points | 2.7 | 36.0 |  | 2.85, 2.67 | 23.6, 37.2 |  | 35.1, 35.4 | 36.5, 36.2 | 73.8, 76.6 | 66.7, 68.6 |  | 134, 133 | 662, 774 | 151, 147 |  | 2.71, 2.86 | 35.5, 33.8 |  | 35.3, 35.3 | 36.1, 36.6 | 58.0, 60.1 | 51.6, 53.5 |  | 166, 164 | 738, 729 | 197, 196 |
|  |  | 48.0 |  | 2.83, 2.70 | 23.9, 36.0 |  | 46.6, 47.1 | 47.9, 47.7 | 84.5, 83.1 | 78.4, 77.7 |  | 115, 102 | 664, 759 | 128, 111 |  | 2.75, 2.85 | 33.5, 33.7 |  | 46.8, 47.1 | 47.7, 47.8 | 67.3, 68.7 | 61.2, 63.5 |  | 181, 147 | 723, 727 | 220, 171 |
|  |  | 60.0 |  | 2.76, 2.72 | 28.4, 35.4 |  | 58.0, 58.5 | 59.4, 59.2 | 90.0, 83.6 | 84.9, 76.2 |  | 112, 222 | 699, 745 | 123, 267 |  | 2.62, 2.80 | 35.3, 34.8 |  | 58.3, 58.6 | 59.0, 59.6 | 73.3, 74.8 | 68.1, 69.9 |  | 212, 188 | 735, 733 | 266, 229 |
|  |  | 72.0 |  | 2.89, 2.61 | 33.7, 32.5 |  | 69.6, 70.0 | 70.6, 70.6 | 93.5, 90.2 | 88.6, 85.4 |  | 157, 163 | 736, 725 | 178, 187 |  | 2.71, 2.59 | 36.3, 35.9 |  | 70.1, 70.4 | 70.6, 70.5 | 81.2, 81.3 | 76.2, 76.8 |  | 301, 255 | 744, 742 | 422, 336 |
|  | 4.5 | 36.0 |  | 4.54, 4.24 | 25.0, 37.7 |  | 35.2, 35.6 | 37.3, 36.8 | 84.5, 83.7 | 77.5, 78.1 |  | 163, 122 | 687, 787 | 189, 133 |  | 4.39, 4.66 | 34.3, 35.0 |  | 35.7, 35.4 | 37.3, 37.0 | 65.8, 71.5 | 58.7, 66.8 |  | 219, 123 | 739, 753 | 277, 139 |
|  |  | 48.0 |  | 4.37, 4.24 | 24.4, 37.4 |  | 46.6, 46.9 | 48.4, 48.0 | 89.8, 91.3 | 84.0, 87.1 |  | 148, 99 | 673, 778 | 168, 106 |  | 4.50, – | 36.4, – |  | 46.8, – | 48.2, – | 73.3, – | 68.5, – |  | 168, – | 751, – | 199, – |
|  |  | 60.0 |  | 4.59, 4.20 | 26.5, 36.4 |  | 58.1, 58.6 | 59.6, 60.2 | 102.6, 91.2 | 99.6, 86.0 |  | 76, 172 | 694, 760 | 80, 197 |  | 4.38, 4.43 | 33.4, 36.1 |  | 58.4, 58.7 | 59.7, 59.7 | 80.4, 83.5 | 74.9, 79.9 |  | 242, 131 | 729, 752 | 316, 149 |
|  |  | 72.0 |  | 4.46, 4.38 | 28.6, 33.8 |  | 69.5, 70.2 | 71.3, 71.5 | 108.9, 104.2 | 106.8, 102.2 |  | 60, 62 | 710, 746 | 63, 65 |  | 4.40, 4.23 | 35.8, 33.5 |  | 70.0, 70.3 | 71.1, 71.2 | 88.8, 88.2 | 84.4, 83.4 |  | 221, 254 | 747, 730 | 279, 337 |
| New Supplem-entary points | 2.7 | 54.0 |  | 2.86 | 34.9 |  | 52.9 | 53.7 | 73.8 | 67.0 |  | 263 | 735 | 329 |  | 3.02 | 35.5 |  | 52.9 | 53.7 | 72.4 | 66.8 |  | 192 | 739 | 234 |
|  | 2.96 | 41.3 |  | 3.15 | 36.2 |  | 40.6 | 41.3 | 72.9 | 66.2 |  | 171 | 755 | 196 |  | 3.24 | 37.3 |  | 40.7 | 41.5 | 65.9 | 59.5 |  | 177 | 754 | 212 |
|  |  | 66.7 |  | 3.10 | 35.4 |  | 65.2 | 66.2 | 87.1 | 81.0 |  | 238 | 745 | 290 |  | 3.17 | 36.3 |  | 65.3 | 66.0 | 79.8 | 74.9 |  | 245 | 745 | 319 |
|  | 3.6 | 36.0 |  | 3.36 | 35.9 |  | 35.5 | 36.5 | 71.2 | 64.9 |  | 153 | 759 | 172 |  | 3.86 | 38.9 |  | 35.3 | 36.6 | 64.9 | 59.6 |  | 143 | 772 | 164 |
|  |  | 54.0 |  | 3.84 | 35.9 |  | 52.9 | 53.9 | 83.6 | 78.0 |  | 182 | 753 | 210 |  | 3.90 | 38.3 |  | 53.1 | 54.0 | 76.4 | 70.9 |  | 195 | 762 | 239 |
|  |  | 54.0 |  | 3.70 | 35.6 |  | 53.0 | 53.8 | 85.4 | 80.1 |  | 155 | 753 | 175 |  | 3.92 | 35.3 |  | 52.9 | 54.1 | 74.1 | 69.2 |  | 195 | 740 | 239 |
|  |  | 72.0 |  | 3.76 | 35.6 |  | 70.5 | 71.2 | 96.0 | 90.6 |  | 207 | 752 | 246 |  | 3.59 | 35.3 |  | 70.5 | 71.3 | 86.6 | 82.3 |  | 209 | 742 | 261 |
|  | 4.25 | 41.3 |  | 4.36 | 34.3 |  | 40.6 | 41.3 | 77.6 | 70.8 |  | 203 | 747 | 240 |  | 4.56 | 37.2 |  | 40.8 | 41.9 | 71.3 | 65.0 |  | 195 | 760 | 239 |
|  |  | 66.7 |  | 4.40 | 35.5 |  | 65.3 | 66.4 | 92.4 | 86.4 |  | 258 | 750 | 319 |  | 4.35 | 37.8 |  | 65.2 | 66.3 | 84.8 | 80.6 |  | 199 | 760 | 244 |
|  | 4.50 | 54.0 |  | 4.64 | 35.9 |  | 53.0 | 53.9 | 82.7 | 76.4 |  | 255 | 752 | 315 |  | 4.53 | 35.6 |  | 53.0 | 54.3 | 78.3 | 73.0 |  | 203 | 746 | 251 |

A_m_ = 6460 mm^2^ for all calculations. A_p_ = 3640 mm^2^ or A_p_ = 4550 mm^2^ for the a* = 0.45 or a* = 0.28 die, respectively. In the original bookend points section, most cells have two entries, with each entry corresponding to a different process replicate; unfortunately, the data for the 2^nd^ process replicate of the a* = 0.28, Ṁ_p_ = 4.5 kg/hr, and T_m_= 48 °C conditions was lost due to corruption of the data during file transfer.

**Table S5** Cumulatively averaged heat transfer coefficients and corresponding data needed for their calculation up to the nominal 2/3 of the die length.

|  |  | |  | a* = 0.45 | | | | | | | | | | |  | a* = 0.28 | | | | | | | | | | |
| --- | --- | --- | --- | --- | --- | --- | --- | --- | --- | --- | --- | --- | --- | --- | --- | --- | --- | --- | --- | --- | --- | --- | --- | --- | --- | --- |
| Experimental series  and process targets | | |  | Measured mass flow rate (kg/hr) | |  | Temperature  (°C) | | | |  | Heat transfer coefficients  (W/m^2^∙k) | | |  | Mass flow rate (kg/hr) | |  | Temperature  (°C) | | | |  | Heat transfer coefficients  (W/m^2^∙k) | | |
| Series | Ṁ_p_  (kg/hr) | T_m_  (°C) |  | Ṁ_p_ | Ṁ_m_ |  | T_m,i_ | T_m,o_ | T_p,i_ | T_p,o_ |  | U_p_ | h_m_ | h_p_ |  | Ṁ_p_ | Ṁ_m_ |  | T_m,i_ | T_m,o_ | T_p,i_ | T_p,o_ |  | U_p_ | h_m_ | h_p_ |
| Original bookend points | 2.7 | 36.0 |  | 2.85, 2.67 | 23.6, 37.2 |  | 36.4, 36.2 | 43.9, 41.1 | 150.2, 149.5 | 75.2, 78.1 |  | 347, 297 | 696, 814 | 495, 380 |  | 2.71, 2.86 | 35.5, 33.8 |  | 36.2, 36.3 | 41.7, 42.1 | 148.6, 148.3 | 59.2, 61.3 |  | 384, 385 | 792, 779 | 605, 611 |
|  |  | 48.0 |  | 2.83, 2.70 | 23.9, 36.0 |  | 47.8, 47.7 | 54.4, 52.4 | 149.5, 147.9 | 85.7, 84.2 |  | 317, 305 | 690, 790 | 437, 397 |  | 2.75, 2.85 | 33.5, 33.7 |  | 47.7, 47.8 | 52.9, 53.2 | 148.3, 148.2 | 68.5, 69.7 |  | 389, 390 | 765, 767 | 630, 632 |
|  |  | 60.0 |  | 2.76, 2.72 | 28.4, 35.4 |  | 59.2, 59.1 | 64.5, 63.5 | 148.6, 147.3 | 91.0, 85.0 |  | 322, 375 | 722, 775 | 440, 528 |  | 2.62, 2.80 | 35.3, 34.8 |  | 59.1, 59.4 | 63.6, 64.0 | 147.2, 147.5 | 74.4, 75.7 |  | 413, 426 | 770, 766 | 692, 730 |
|  |  | 72.0 |  | 2.89, 2.61 | 33.7, 32.5 |  | 70.6, 70.8 | 74.2, 74.5 | 147.9, 146.2 | 94.5, 91.1 |  | 380, 386 | 758, 747 | 545, 559 |  | 2.71, 2.59 | 36.3, 35.9 |  | 70.8, 71.1 | 74.6, 74.8 | 147.1, 147.6 | 82.2, 82.2 |  | 465, 448 | 772, 770 | 848, 796 |
|  | 4.5 | 36.0 |  | 4.54, 4.24 | 25.0, 37.7 |  | 37.0, 36.8 | 45.5, 42.3 | 142.6, 142.9 | 85.9, 84.8 |  | 399, 377 | 710, 816 | 602, 522 |  | 4.39, 4.66 | 34.3, 35.0 |  | 37.2, 36.9 | 44.3, 43.8 | 143.8, 143.9 | 67.1, 72.5 |  | 505, 464 | 781, 790 | 980, 830 |
|  |  | 48.0 |  | 4.37, 4.24 | 24.4, 37.4 |  | 48.4, 48.1 | 56.3, 53.5 | 143.9, 144.1 | 90.9, 92.1 |  | 404, 372 | 693, 801 | 621, 517 |  | 4.50, – | 36.4, – |  | 48.3, – | 54.4, – | 143.5, – | 74.2, – |  | 529, – | 785, – | 1071, – |
|  |  | 60.0 |  | 4.59, 4.20 | 26.5, 36.4 |  | 59.8, 59.6 | 65.8, 64.8 | 142.3, 142.5 | 103.2, 92.2 |  | 336, 444 | 707, 782 | 470, 673 |  | 4.38, 4.43 | 33.4, 36.1 |  | 59.7, 59.9 | 65.4, 65.3 | 142.7, 143.5 | 81.5, 84.3 |  | 532, 495 | 756, 777 | 1128, 950 |
|  |  | 72.0 |  | 4.46, 4.38 | 28.6, 33.8 |  | 71.3, 71.5 | 75.7, 75.7 | 142.8, 142.2 | 109.4, 104.6 |  | 318, 376 | 720, 760 | 433, 535 |  | 4.40, 4.23 | 35.8, 33.5 |  | 71.3, 71.5 | 75.9, 76.4 | 142.7, 144.3 | 89.6, 89.2 |  | 543, 545 | 769, 752 | 1156, 1193 |
| New Supplem-entary points | 2.7 | 54.0 |  | 2.86 | 34.9 |  | 53.4 | 57.4 | 144.9 | 75.1 |  | 459 | 771 | 716 |  | 3.02 | 35.5 |  | 53.7 | 58.4 | 144.7 | 73.5 |  | 414 | 774 | 692 |
|  | 2.96 | 41.3 |  | 3.15 | 36.2 |  | 41.2 | 46.0 | 143.9 | 74.3 |  | 399 | 794 | 571 |  | 3.24 | 37.3 |  | 41.5 | 46.8 | 144.5 | 67.1 |  | 404 | 798 | 652 |
|  |  | 66.7 |  | 3.10 | 35.4 |  | 65.8 | 69.5 | 145.5 | 88.3 |  | 440 | 771 | 669 |  | 3.17 | 36.3 |  | 66.0 | 70.1 | 144.4 | 80.7 |  | 477 | 774 | 889 |
|  | 3.6 | 36.0 |  | 3.36 | 35.9 |  | 36.6 | 42.0 | 144.4 | 72.5 |  | 414 | 799 | 601 |  | 3.86 | 38.9 |  | 36.4 | 42.5 | 145.5 | 65.9 |  | 453 | 817 | 775 |
|  |  | 54.0 |  | 3.84 | 35.9 |  | 53.5 | 58.3 | 145.2 | 84.7 |  | 465 | 782 | 723 |  | 3.90 | 38.3 |  | 53.7 | 59.1 | 144.5 | 77.4 |  | 473 | 796 | 854 |
|  |  | 54.0 |  | 3.70 | 35.6 |  | 53.5 | 58.3 | 145.1 | 86.5 |  | 425 | 781 | 632 |  | 3.92 | 35.3 |  | 54.1 | 59.6 | 146.1 | 75.1 |  | 524 | 773 | 1065 |
|  |  | 72.0 |  | 3.76 | 35.6 |  | 71.3 | 74.9 | 144.9 | 97.1 |  | 443 | 772 | 676 |  | 3.59 | 35.3 |  | 71.4 | 75.8 | 144.8 | 87.5 |  | 493 | 766 | 955 |
|  | 4.25 | 41.3 |  | 4.36 | 34.3 |  | 41.4 | 47.9 | 144.0 | 78.9 |  | 496 | 780 | 802 |  | 4.56 | 37.2 |  | 41.9 | 48.0 | 145.4 | 72.5 |  | 501 | 799 | 947 |
|  |  | 66.7 |  | 4.40 | 35.5 |  | 66.3 | 70.5 | 145.5 | 93.6 |  | 529 | 773 | 898 |  | 4.35 | 37.8 |  | 66.4 | 71.1 | 144.4 | 85.6 |  | 551 | 786 | 1165 |
|  | 4.50 | 54.0 |  | 4.64 | 35.9 |  | 53.8 | 59.2 | 145.9 | 83.9 |  | 586 | 781 | 1068 |  | 4.53 | 35.6 |  | 54.2 | 60.3 | 144.9 | 79.4 |  | 527 | 776 | 1075 |

A_m_ = 12,920 mm^2^ for all calculations. A_p_ = 7,760 mm^2^ or A_p_ = 9,700 mm^2^ for the a* = 0.45 or a* = 0.28 die, respectively. In the original bookend points section, most cells have two entries, with each entry corresponding to a different process replicate; unfortunately, the data for the 2^nd^ process replicate of the a* = 0.28, Ṁ_p_ = 4.5 kg/hr, and T_m_= 48 °C conditions was lost due to corruption of the data during file transfer.

**Table S6** Cumulatively averaged heat transfer coefficients and corresponding data needed for their calculation for the entire die length.

|  |  | |  | a* = 0.45 | | | | | | | | | | |  | a* = 0.28 | | | | | | | | | | |
| --- | --- | --- | --- | --- | --- | --- | --- | --- | --- | --- | --- | --- | --- | --- | --- | --- | --- | --- | --- | --- | --- | --- | --- | --- | --- | --- |
| Experimental series  and process targets | | |  | Measured mass flow rate (kg/hr) | |  | Temperature  (°C) | | | |  | Heat transfer coefficients  (W/m^2^∙k) | | |  | Mass flow rate (kg/hr) | |  | Temperature  (°C) | | | |  | Heat transfer coefficients  (W/m^2^∙k) | | |
| Series | Ṁ_p_  (kg/hr) | T_m_  (°C) |  | Ṁ_p_ | Ṁ_m_ |  | T_m,i_ | T_m,o_ | T_p,i_ | T_p,o_ |  | U_p_ | h_m_ | h_p_ |  | Ṁ_p_ | Ṁ_m_ |  | T_m,i_ | T_m,o_ | T_p,i_ | T_p,o_ |  | U_p_ | h_m_ | h_p_ |
| Original bookend points | 2.7 | 36.0 |  | 2.85, 2.67 | 23.6, 37.2 |  | 35.1, 35.4 | 43.9, 41.1 | 150.2, 149.5 | 66.7, 68.6 |  | 271, 239 | 694, 811 | 358, 292 |  | 2.71, 2.86 | 35.5, 33.8 |  | 35.3, 35.3 | 41.7, 42.1 | 148.6, 148.3 | 51.6, 53.5 |  | 306, 305 | 789, 776 | 437, 438 |
|  |  | 48.0 |  | 2.83, 2.70 | 23.9, 36.0 |  | 46.6, 47.1 | 54.4, 52.4 | 149.5, 147.9 | 78.4, 77.7 |  | 245, 234 | 688, 788 | 314, 286 |  | 2.75, 2.85 | 33.5, 33.7 |  | 46.8, 47.1 | 52.9, 53.2 | 148.3, 148.2 | 61.2, 63.5 |  | 314, 303 | 762, 764 | 460, 438 |
|  |  | 60.0 |  | 2.76, 2.72 | 28.4, 35.4 |  | 58.0, 58.5 | 64.5, 63.5 | 148.6, 147.3 | 84.9, 76.2 |  | 247, 321 | 721, 772 | 313, 432 |  | 2.62, 2.80 | 35.3, 34.8 |  | 58.3, 58.6 | 63.6, 64.0 | 147.2, 147.5 | 68.1, 69.9 |  | 339, 338 | 768, 764 | 516, 514 |
|  |  | 72.0 |  | 2.89, 2.61 | 33.7, 32.5 |  | 69.6, 70.0 | 74.2, 74.5 | 147.9, 146.2 | 88.6, 85.4 |  | 299, 306 | 756, 745 | 397, 409 |  | 2.71, 2.59 | 36.3, 35.9 |  | 70.1, 70.4 | 74.6, 74.8 | 147.1, 147.6 | 76.2, 76.8 |  | 402, 376 | 770, 768 | 676, 606 |
|  | 4.5 | 36.0 |  | 4.54, 4.24 | 25.0, 37.7 |  | 35.2, 35.6 | 45.5, 42.3 | 142.6, 142.9 | 77.5, 78.1 |  | 315, 286 | 708, 814 | 434, 366 |  | 4.39, 4.66 | 34.3, 35.0 |  | 35.7, 35.4 | 44.3, 43.8 | 143.8, 143.9 | 58.7, 66.8 |  | 401, 340 | 778, 788 | 666, 511 |
|  |  | 48.0 |  | 4.37, 4.24 | 24.4, 37.4 |  | 46.6, 46.9 | 56.3, 53.5 | 143.9, 144.1 | 84.0, 87.1 |  | 312, 275 | 691, 800 | 432, 349 |  | 4.50, – | 36.4, – |  | 46.8, – | 54.4, – | 143.5, – | 68.5, – |  | 397, – | 783, – | 654, – |
|  |  | 60.0 |  | 4.59, 4.20 | 26.5, 36.4 |  | 58.1, 58.6 | 65.8, 64.8 | 142.3, 142.5 | 99.6, 86.0 |  | 242, 346 | 706, 780 | 307, 478 |  | 4.38, 4.43 | 33.4, 36.1 |  | 58.4, 58.7 | 65.4, 65.3 | 142.7, 143.5 | 74.9, 79.9 |  | 425, 363 | 754, 776 | 753, 569 |
|  |  | 72.0 |  | 4.46, 4.38 | 28.6, 33.8 |  | 69.5, 70.2 | 75.7, 75.7 | 142.8, 142.2 | 106.8, 102.2 |  | 224, 262 | 720, 759 | 277, 334 |  | 4.40, 4.23 | 35.8, 33.5 |  | 70.0, 70.3 | 75.9, 76.4 | 142.7, 144.3 | 84.4, 83.4 |  | 423, 437 | 768, 750 | 737, 795 |
| New Supplem-entary points | 2.7 | 54.0 |  | 2.86 | 34.9 |  | 52.9 | 57.4 | 144.9 | 67.0 |  | 389 | 768 | 567 |  | 3.02 | 35.5 |  | 52.9 | 58.4 | 144.7 | 66.8 |  | 334 | 772 | 501 |
|  | 2.96 | 41.3 |  | 3.15 | 36.2 |  | 40.6 | 46.0 | 143.9 | 66.2 |  | 319 | 790 | 425 |  | 3.24 | 37.3 |  | 40.7 | 46.8 | 144.5 | 59.5 |  | 323 | 795 | 471 |
|  |  | 66.7 |  | 3.10 | 35.4 |  | 65.2 | 69.5 | 145.5 | 81.0 |  | 367 | 768 | 521 |  | 3.17 | 36.3 |  | 65.3 | 70.1 | 144.4 | 74.9 |  | 393 | 771 | 647 |
|  | 3.6 | 36.0 |  | 3.36 | 35.9 |  | 35.5 | 42.0 | 144.4 | 64.9 |  | 321 | 796 | 428 |  | 3.86 | 38.9 |  | 35.3 | 42.5 | 145.5 | 59.6 |  | 341 | 815 | 505 |
|  |  | 54.0 |  | 3.84 | 35.9 |  | 52.9 | 58.3 | 145.2 | 78.0 |  | 365 | 780 | 514 |  | 3.90 | 38.3 |  | 53.1 | 59.1 | 144.5 | 70.9 |  | 374 | 793 | 590 |
|  |  | 54.0 |  | 3.70 | 35.6 |  | 53.0 | 58.3 | 145.1 | 80.1 |  | 330 | 779 | 447 |  | 3.92 | 35.3 |  | 52.9 | 59.6 | 146.1 | 69.2 |  | 403 | 771 | 676 |
|  |  | 72.0 |  | 3.76 | 35.6 |  | 70.5 | 74.9 | 144.9 | 90.6 |  | 359 | 770 | 505 |  | 3.59 | 35.3 |  | 70.5 | 75.8 | 144.8 | 82.3 |  | 389 | 764 | 642 |
|  | 4.25 | 41.3 |  | 4.36 | 34.3 |  | 40.6 | 47.9 | 144.0 | 70.8 |  | 393 | 777 | 573 |  | 4.56 | 37.2 |  | 40.8 | 48.0 | 145.4 | 65.0 |  | 392 | 796 | 632 |
|  |  | 66.7 |  | 4.40 | 35.5 |  | 65.3 | 70.5 | 145.5 | 86.4 |  | 431 | 771 | 658 |  | 4.35 | 37.8 |  | 65.2 | 71.1 | 144.4 | 80.6 |  | 421 | 784 | 721 |
|  | 4.50 | 54.0 |  | 4.64 | 35.9 |  | 53.0 | 59.2 | 145.9 | 76.4 |  | 469 | 779 | 748 |  | 4.53 | 35.6 |  | 53.0 | 60.3 | 144.9 | 73.0 |  | 409 | 774 | 692 |

A_m_ = 19,380 mm^2^ for all calculations. A_p_ = 12,000 mm^2^ or A_p_ = 15,000 mm^2^ for the a* = 0.45 or a* = 0.28 die, respectively. In the original bookend points section, most cells have two entries, with each entry corresponding to a different process replicate; unfortunately, the data for the 2^nd^ process replicate of the a* = 0.28, Ṁ_p_ = 4.5 kg/hr, and T_m_= 48 °C conditions was lost due to corruption of the data during file transfer.


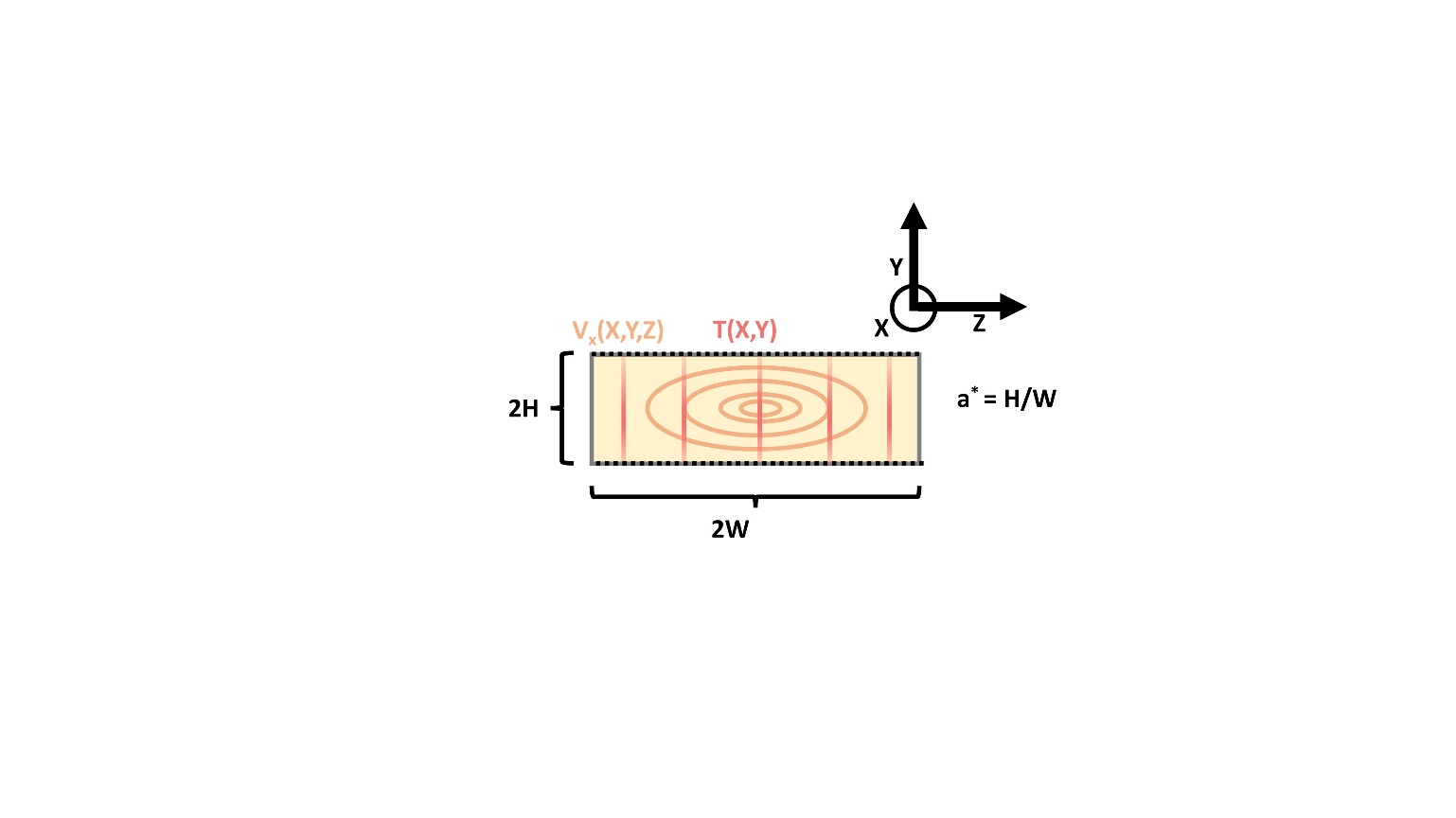

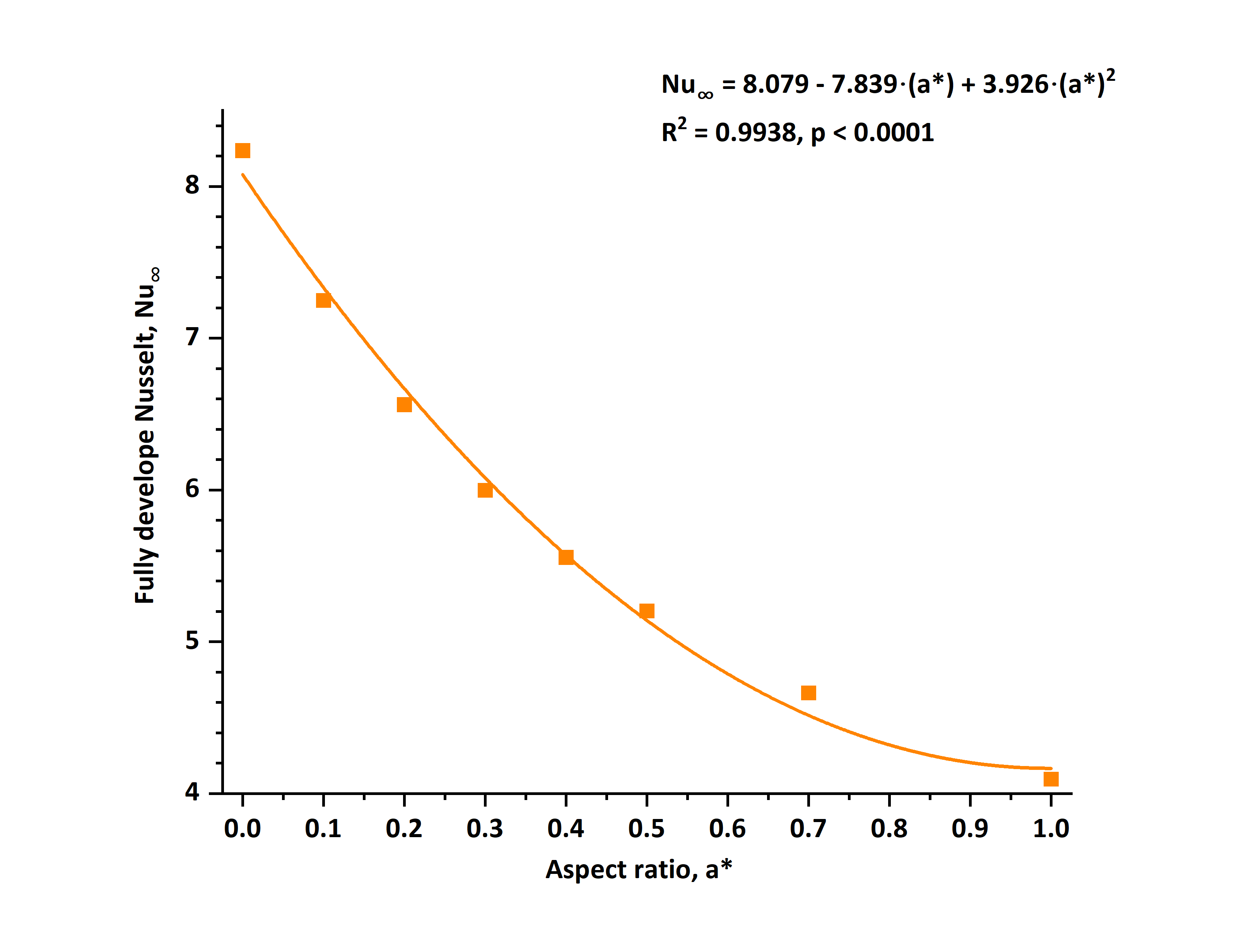


k_wall, x_ ~ 0

Q̇_y_ = constant

Q̇_y_ = constant

Q̇_z_ = 0

Q̇_z_ = 0

**Figure S2** Fully developed Nusselt numbers (Nu_∞_) for rectangular duct aspect ratios (a*) from Shah and London (1978). The short sides of the duct are treated adiabatically (Q̇_z_ = 0) while the top and bottom have equal and constant heat fluxes through the duct wall (Q̇_y_ = constant) and negligibly important axial conduction within the wall (K_wall, x_ ~ 0); This boundary condition can be referenced as the H1 scenario as per Shah and London (1978). (See table 44, page 206). The discrete data points were modeled as a 2^nd^ order polynomial so that exact values of Nu_∞_ could be calculated for this work.
